# Real-Time Monitoring of the Yeast Intracellular State During Bioprocesses With a Toolbox of Biosensors

**DOI:** 10.3389/fmicb.2021.802169

**Published:** 2022-01-07

**Authors:** Luca Torello Pianale, Peter Rugbjerg, Lisbeth Olsson

**Affiliations:** ^1^Industrial Biotechnology Division, Department of Biology and Biological Engineering, Chalmers University of Technology, Gothenburg, Sweden; ^2^Enduro Genetics ApS, Copenhagen, Denmark

**Keywords:** fluorescence, stress, ATP concentration, oxidative stress, intracellular pH (pHi), glycolytic flux, ribosome production

## Abstract

Industrial fermentation processes strive for high robustness to ensure optimal and consistent performance. Medium components, fermentation products, and physical perturbations may cause stress and lower performance. Cellular stress elicits a range of responses, whose extracellular manifestations have been extensively studied; whereas intracellular aspects remain poorly known due to lack of tools for real-time monitoring. Genetically encoded biosensors have emerged as promising tools and have been used to improve microbial productivity and tolerance toward industrially relevant stresses. Here, fluorescent biosensors able to sense the yeast intracellular environment (pH, ATP levels, oxidative stress, glycolytic flux, and ribosome production) were implemented into a versatile and easy-to-use toolbox. Marker-free and efficient genome integration at a conserved site on chromosome X of Saccharomyces cerevisiae strains and a commercial Saccharomyces boulardii strain was developed. Moreover, multiple biosensors were used to simultaneously monitor different intracellular parameters in a single cell. Even when combined together, the biosensors did not significantly affect key physiological parameters, such as specific growth rate and product yields. Activation and response of each biosensor and their interconnection were assessed using an advanced micro-cultivation system. Finally, the toolbox was used to screen cell behavior in a synthetic lignocellulosic hydrolysate that mimicked harsh industrial substrates, revealing differences in the oxidative stress response between laboratory (CEN.PK113-7D) and industrial (Ethanol Red) S. cerevisiae strains. In summary, the toolbox will allow both the exploration of yeast diversity and physiological responses in natural and complex industrial conditions, as well as the possibility to monitor production processes.

## Introduction

Industrial fermentation processes use microorganisms as cell factories to convert a given substrate to valuable products (Demain, 2000). However, complex substrates (e.g., lignocellulosic hydrolysates), product inhibition (e.g., ethanol), and other perturbations (e.g., inhibitors or physical constrains) are stressful for the cells, leading to suboptimal production (Deparis et al., 2017). Although efforts have been made to develop microbial strains more tolerant to the stressors present during different fermentation processes (Swinnen et al., 2017; Ko et al., 2020; Liu et al., 2020), achieving this at industrial scale remains a challenge (Wehrs et al., 2019). Because controlled laboratory conditions cannot fully mimic industrial settings, new strains tend to perform poorly upon scaling up (Wehrs et al., 2019). Moreover, while the extracellular environment and phenotypic characteristics of microorganisms (e.g., titers, rates, and yields) are easily analyzed online or via real-time sampling, little is known about the microbes’ intracellular and metabolic responses in these complex environments. This discrepancy in information comes from the lack of tools to monitor parameters, such as intracellular pH, ATP concentration, and oxidative stress. Understanding the cellular responses and linking them to specific environmental conditions would lead to more robust strains and consistent production processes.

Genetically encoded fluorescent biosensors are promising tools for evaluating the intracellular environment, as they can sense compounds or conditions inside the cell, and thus track the ensuing response (Carpenter et al., 2018). They have already been used both to improve microbial production (Raman et al., 2014), such as in the case of muconic and octanoic acids (Leavitt et al., 2017; Wang et al., 2020; Baumann et al., 2021), and tolerance to industrially relevant stresses (Alvarez-Gonzalez and Dixon, 2019). However, some constraints limited the application of biosensors in real-time strain or process diagnostics. For example, acidic environments, often found in bioprocesses, should be taken into account when choosing a fluorescent protein as pH affects the fluorescence output (Shinoda et al., 2018). Combining multiple fluorescent proteins in the same cell is hampered by overlap of excitation/emission spectra (Botman et al., 2019). Tagging up to four different proteins in separate organelles with four distinct fluorescent probes was shown not to cause spectral overlap or organelle malfunction (Higuchi-Sanabria et al., 2016). A more recent study confirmed the possibility of tagging multiple proteins in different organelles with non-overlapping fluorescent probes, although it led to some protein disfunctions (Zhu et al., 2019). In Escherichia coli, efforts have been made to develop a platform for real-time detection of metabolites using fluorescent proteins (Rogers et al., 2015). However, testing and monitoring the physiological performance of yeasts with biosensors remain uncommon for the risk of affecting productivity, particularly if multiple biosensors are combined in the same cell.

Saccharomyces cerevisiae is one of the most studied and used microorganisms in the laboratory and bioindustry (Kampranis and Makris, 2012). Owing to its wide range of applications, industrial strains of S. cerevisiae have been developed to address specific requirements, such as higher ethanol or biomass production (Parapouli et al., 2020). Moreover, S. cerevisiae strains with interesting features for industrial purposes are being isolated from natural habitats (da Conceição et al., 2015; Liti, 2015). In spite of this wide diversity, only an accurate assessment of cell physiology and the intracellular environment will reveal the mechanisms responsible for greater tolerance and robustness and, hence, drive a more targeted, faster, and cost-effective development of industrial strains (Molinet and Cubillos, 2020).

**FIGURE 1:**
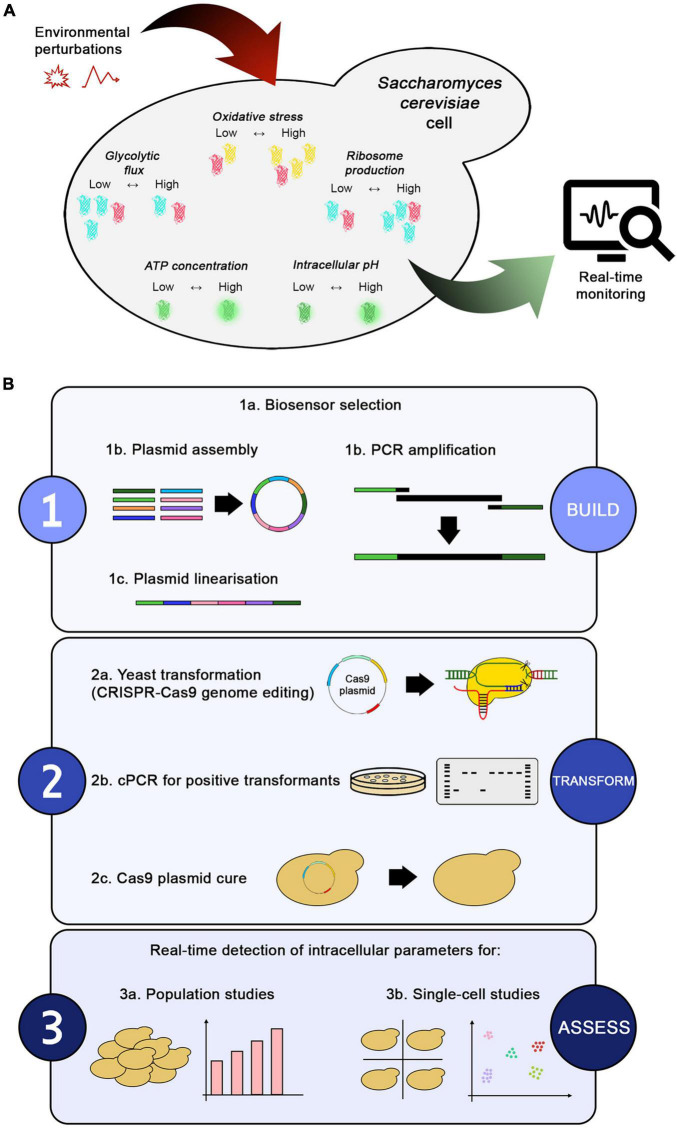
Toolbox overview and workflow. **(A)** Environmental perturbations in a bioprocess directly affect the intracellular environment of a yeast cell. The biosensors present in the toolbox enable real-time monitoring of changes in intracellular parameters, such as glycolytic flux (with GlyRNA), oxidative stress (with OxPro), ribosome production (with RibPro), intracellular pH (with sfpHluorin), and ATP concentration (with QUEEN-2m). **(B)** The workflow for generating the toolbox can be summarized in three steps: (1) building the desired biosensor, (2) transforming yeast, and (3) assessing the biosensors’ output under different conditions. Once selected, the biosensors can be assembled into plasmids or amplified by PCR to obtain marker-free constructs used for genome integration via CRISPR-Cas9 editing technology. Positive transformants are confirmed by colony PCR, followed by curing of the Cas9 plasmid to remove the selection markers. Finally, the new strains can be used for population or single-cell studies. Alternative plasmid assembly or genome integration methods can be easily fitted in the workflow.

The present study aimed to select various genetically encoded fluorescent biosensors capable of sensing key intracellular parameters (e.g., pH, ATP concentration, ribosome production, oxidative stress, and glycolytic flux) and implement them in a toolbox for real-time monitoring of yeast strains (Figure 1 and Table 1). Owing to its easy and versatile build-transform-assess workflow (Figure 1B), this toolbox could facilitate the exploration of yeast metabolism during industrial processes and help monitor production. First, we demonstrated that the toolbox could be integrated in the genome by an easy, efficient, and marker-free method, compatible with different S. cerevisiae strains. Second, we showed that the selected biosensors did not affect key yeast performance indicators of growth and metabolism, thus proving reliable monitoring of the intracellular state. Third, we demonstrated the simultaneous function of multiple biosensors in the same cell by using non-overlapping fluorescence spectra. Finally, we applied the toolbox for real-time monitoring under stressful conditions mimicking industrial fermentation and demonstrated that different S. cerevisiae strains elicited different stress responses.

**TABLE 1:** Summary of biosensors in the toolbox.

Biosensor	Intracellular parameter	Detection of	Fluorescent protein (s)	Optimal λex (nm)	Optimal λem (nm)	References
QUEEN-2m	ATP concentration	ATP	QUEEN-2m	410 and 480	520	Takaine et al., 2019
sfpHluorin	Intracellular pH	pH	SfpHluorin	390 and 470	512	Reifenrath and Boles, 2018
GlyRNA	Glycolytic flux	Fructose-bisphosphate	mTurquoise2	436	488	Ortega et al., 2021
			mCherry*	587	610	
OxPro	Oxidative stress response	YAP1 activation	YmYPET	516	526	Zhang et al., 2016
			mCherry*	587	610	
RibPro	Ribosome production	RPL13A production	mTurquoise2	436	488	Janssens and Veenhoff, 2016
			mCherry*	587	610	

List of the biosensors included in the toolbox and characterization of the fluorescent proteins used in this study. *Refers to a fluorescent protein used for normalization, not for the detection of the intracellular parameter.

## Materials and Methods

### Strains and Media Composition

All yeast strains used in this study are listed in Table 2. The S. cerevisiae strains bearing fluorescent biosensors were constructed from the laboratory strain CEN.PK113-7D (MATa URA3 HIS3 LEU2 TRP1 MAL2-8c SUC2) (Entian and Kötter, 2007) and the commercial bioethanol-producing strain Ethanol Red (Société Industrielle Lesaffre, Division Leaf). Chemically competent E. coli DH5α were used for plasmid construction and selection (Seidman and Struhl, 1998).

**TABLE 2:** Yeast strains.

Strain	Description	References
CEN.PK113-7D*	Haploid laboratory strain	Entian and Kötter, 2007
S288C*	Haploid laboratory strain	Mortimer and Johnston, 1986
Ethanol red*	Diploid industrial strain	Société industrielle lesaffre, division leaf
Red star*	Industrial strain	Red star yeast company, LLC
Thermosacc*	Industrial strain	Lallemand biofuels and distilled spirits, United States
PE-2*	Diploid industrial strain	Fermentec, brazil
CCUG 53310*	Industrial strain	Purwadi et al., 2007
LARS*	Industrial strain	Our laboratory collection
X218-1A*	Haploid wild-type strain	Raschke et al., 1973
LBCM1001*, 1003*, 1008*, 1013*, 1014, 1017*, 1030*, 1037*, 1046*, 1067*, 1079*, 1095*, 1097*, 1099*, 1103*, 1106*, 1109*	Wild-type isolates from LBCM collection, which includes 138 strains isolated from cachaça distilleries located in Brazil	da Conceição et al., 2015
Saccharomyces boulardii CNCM I-745	Commercial probiotic strain, purchased from PRECOSA (Biocodex, France)	Moré and Swidsinski, 2015
Saccharomyces bayanus FM361	Wild-type strain	Cliften et al., 2001
Saccharomyces castellii FM476	Wild-type strain	Cliften et al., 2001
Saccharomyces kluyveri FM479	Wild-type strain	Cliften et al., 2001
Saccharomyces kudriavzevii FM527	Wild-type strain	Cliften et al., 2001
Saccharomyces mikatae FM356	Wild-type strain	Cliften et al., 2001
Zygosaccharomyces bailii CBS 1170	Wild-type strain	Suh et al., 2013
Candida intermedia CBS 2044	Wild-type strain	Pham et al., 2011
Kluyveromyces marxianus NCYC 179	Industrial strain	Steele and Miller, 1974

Yeast strains used in this study. *Refers to Saccharomyces cerevisiae strains.

For transformation and for curing the Cas9-bearing plasmid used in genome integration, yeast strains were grown in YPD medium (10 g/L yeast extract, 20 g/L peptone, and 20 g/L glucose, plus 15 g/L agar for plates), supplemented with 200 mg/L G418 sulfate when required. For flask and BioLector I (M2p-labs GmbH) screening, synthetic defined minimal Verduyn (“Delft”) medium adjusted to pH 5 was used. The medium contained 20 g/L glucose, 5 g/L (NH_4_)_2_SO_4_, 3 g/L KH_2_PO_4_, 1 g/L MgSO_4_⋅7H_2_O, 20.4 g/L K-phthalate, 1 mL/L trace metal solution, and 1 mL/L vitamin solution (trace metals and vitamin solution compositions are listed in Supplementary Table 1).

When assessing oxidative stress and intracellular pH under specific stressors typically released during pre-treatment of lignocellulosic hydrolysates, acetic acid (4.5 or 6 g/L), furfural (1 or 3 g/L), vanillin (0.5 g/L), or xylose (20 or 40 g/L) were added to Delft medium.

To mimic wheat-straw hydrolysate, a second-generation bioethanol production substrate, a selection of key compounds at specific concentrations was added to Delft medium (Table 3) and pH was adjusted to 5. To serve as growth substrate, this synthetic wheat-straw hydrolysate (SWSH) was diluted at 50 and 80% using Delft medium without any carbon source.

**TABLE 3:** Composition of synthetic wheat-straw hydrolysate.

Compounds	g/L	References
Mannose	1	López-Abelairas et al., 2013
Glucose	68.8	van Dijk et al., 2019
Xylose	36.4	van Dijk et al., 2019
Arabinose	4	Baroi et al., 2015
Galactose	0.6	López-Abelairas et al., 2013
Acetic acid	4.7	van Dijk et al., 2019
Formic acid	1.2	van Dijk et al., 2019
Levulinic acid	–	van Dijk et al., 2019
Furfural	3	van Dijk et al., 2019
5-(Hydroxymethyl)furfural	0.6	van Dijk et al., 2019
Vanillin	0.03	Almeida et al., 2007

*The amounts refer to 100% medium.*

Competent E. coli DH5α were grown in LB medium (10 g/L bacto-tryptone, 5 g/L yeast extract, and 10 g/L NaCl, plus 15 g/L agar for plates) with the required antibiotic (ampicillin 100 mg/L or neomycin 50 mg/L). Plates were incubated at 30°C, and liquid cultures at 30°C and 220 rpm, in order to limit the possibility of recombination events when repeated regions were presents in the plasmids.

### Cloning, Yeast Transformation, and Gene Amplification

#### Cloning

Plasmids used for genome integration and bearing either the CRISPR-Cas9 system or single/pairs of biosensors were generated using the MoClo Modular Cloning System Plasmid Kit (Lee et al., 2015). Constructs not included in the kit, such as promoters or coding sequences, were ordered from Twist Bioscience^1^ and contained suitable flanking restriction sites that could be excised with Eco31I and Esp3I. Alternatively, they were amplified by PCR and inserted in the entry vector pYTK001 (Supplementary Table 2; Lee et al., 2015). The complete list of plasmids generated in this study is provided in Supplementary Table 3.

Plasmid assembly was carried out by mixing 50 ng of each desired plasmid, 1 μL T4 Ligase Buffer 10 × (Thermo Fisher Scientific), 0.5 μL T4 DNA Ligase (Thermo Fisher Scientific), 0.5 μL FastDigest Esp3I or FastDigest Eco31I (Thermo Fisher Scientific), 0.5 μL dithiothreitol 20 mM (if needed), and MilliQ-H_2_O up to 10 μL. Reactions proceeded as follows: 4 min at 37°C, 40 cycles of 1 min at 37°C, 2 min at 16°C, 4 min at 37°C, and final 10 min at 65°C. Next, 5 μL of the assembly reaction was used to transform competent E. coli DH5α, which were plated on LB agar with suitable antibiotics. White colonies were then verified by colony PCR. The correct clones were cultured overnight in suitable LB medium and the target plasmid was purified using the GeneJET Plasmid Miniprep Kit (Thermo Fisher Scientific).

#### Yeast Transformation

Genome integration in yeast was performed using the LiAc/salmon sperm carrier DNA/polyethylene glycol method (Gietz, 2014) and CRISPR/Cas9 for improved integration efficiency (Akhmetov et al., 2018). The backbone Cas9 plasmid was YN2_1_Cas9_exp, developed in a previous study in our lab (Cámara et al., 2020), in which suitable single guide RNA (sgRNA) was inserted to target the desired sequences (Supplementary Table 3).

The sgRNA-targeting regions were identified using CRISPR-ERA (Liu et al., 2015), Yeast CRISPRi (Smith et al., 2016), and CHOPCHOP (Labun et al., 2019). The following parameters were checked: (i) an ATAC-seq value close to 1; (ii) a nucleosome presence value close to 0; (iii) absence of poly-N and off-targets; (iv) CG content between 40 and 60; and (v) presence of the sgRNA in multiple databases. Single-stranded (forward and reverse) oligonucleotides for sgRNAs were ordered from Eurofins and contained sticky ends suitable for assembly in the YN2_1_Cas9_exp vector (Supplementary Table 4). Double-stranded oligonucleotides were generated by combining 20 μL of each single-stranded oligonucleotide (100 μM) and 10 μL 5 × T4 Ligase Buffer. The mixture was incubated at 98°C for 5 min to denature the oligonucleotides, followed by a gradual decrease of 1°C/30 s over 86 cycles to allow for oligonucleotide annealing.

To insert the sgRNA target sequence in the plasmid, YN2_1_Cas9_exp (∼1 μg) and the annealed sgRNA sequence (0.5 μL of the above reaction) underwent the same MoClo steps as described in section “Cloning” using FastDigest Esp3I.

Prior to transformation in yeast, plasmids harboring the donor DNA were linearized with FastDigest NotI (Thermo Fisher Scientific) for 2 h at 37°C, followed by 5 min at 80°C for enzyme inactivation. For each restriction reaction (20 μL), the following reagents were used: 2 μL 10 × FastDigest Buffer, 1 μL FastDigest NotI, 1 μL Fast AP (Thermo Fisher Scientific), ∼1.5 μg of the desired plasmid, and MilliQ-H_2_O up to 20 μL. The restriction reaction was then mixed with 500 ng of suitable Cas9 plasmid (YN2_1_LT58 or YN2_1_LT84), 5 μL salmon sperm DNA (10 mg/mL), and MilliQ-H_2_O up to 75 μL, and used for subsequent yeast transformation. In the case of RPL13A tagging, plasmid pYTK032 bearing mTurquoise2 (Lee et al., 2015) was used as template for PCR amplification of the donor DNA using oligos LT174_F and LT174_R. In this case, 1 μg of purified PCR product was used in the transformation mixture.

Transformation was carried out as described previously (Gietz, 2014), with an 18-min heat-shock. Cells were plated on YPD + G418 plates and incubated for 3 days at 30°C. Colonies were then verified by colony PCR using oligos LT183_F and LT183_R for integration at the X2 site, or LT87 and LT88 for RPL13A tagging (Supplementary Table 4). Positive clones were re-streaked twice on YPD plates with no antibiotic to cure the Cas9 plasmid.

To test genome integration efficiency, the linearized plasmid LT1_33 (pTEFmut8-mCherry) was used as donor DNA. Ten colonies from each strain were tested by colony PCR using oligos LT183_F and LT183_R.

#### PCR and Sequencing

The PCR oligos used in the present study are listed in Supplementary Table 4. All PCR and colony PCR products were amplified as instructed by the manufacturer. Phusion High-Fidelity DNA Polymerase (Thermo Fisher Scientific) was used in 50-μL reactions to amplify constructs to be used for cloning the plasmids or the X2 site to be sent for sequencing (colony PCR). Phire Hot Start II DNA Polymerase (Thermo Fisher Scientific) was used in 20-μL reactions to verify either successful cloning in bacteria or genome integration in yeast. When performing colony PCR, a small lump of cells from the selected colony was diluted in 20 μL MilliQ-H_2_O, microwaved for 5 min at 800 W (yeast colonies only), and 1 μL of the solution was used as template. The PCR products were run on 1% agarose gels, with 0.5 × TAE buffer, and at 80 mV for 40 min. GeneRuler 1 kb DNA Ladder (Thermo Fisher Scientific) was used to estimate product length. When required, PCR products were purified using the GeneJET PCR Purification Kit (Thermo Fisher Scientific).

The X2 fragment from various strains was amplified with oligos LT185_F and LT185_R and sent for sequencing to Macrogen.^2^ Sequences were then aligned for comparison.

### Cultivation Conditions and Analytical Methods

#### Cultivation in Flasks and High-Performance Liquid Chromatography

For strain characterization, a two-step preculture was employed. Specifically, cells were inoculated from a cyostock in 5 mL Delft medium and grown in a 50-mL tube for 24 h. Then, 100 μL were re-inoculated into 10 mL Delft medium and incubated in 100-mL baffled flasks for 16 h. The characterization was performed in 500-mL screw-top shake flasks (Duran), with a one-way valve for CO_2_ release and a swabable valve for sterile sampling connected to the cap (Eppendorf). The working volume was 150 mL, initial optical density at 600 nm (OD_600_) was 0.1, rotation was 140 rpm, and temperature was set to 30°C. N_2_ was flushed for 10 s after cell inoculation to create a microaerobic environment. OD_600_ was measured every 2 h using 1-mL samples. Additional 1-mL samples were taken at 0, 12, and 15 h for yields determination and were centrifuged at 4,000 rpm for 5 min. The supernatant was filtered through 0.2-μm nylon membrane filters (VWR) and extracellular metabolites (glucose, ethanol, acetic acid, and glycerol) were analyzed using a high-performance liquid chromatography system equipped with a refractive index detector (Jasco) and a Rezex ROA-Organic Acid H^+^ column (Phenomenex). Separation was carried out at a flow rate of 0.8 mL/min, 80°C, and using 5 mM H_2_SO_4_ as eluent. The pellet was used for cell dry weight determination upon resuspension in 1 mL distilled water and filtration through a pre-dried and weighed 0.45-μm polyether sulfone membrane (Sartorius). The membrane was dried for 24 h at 70°C and the weight checked again.

Biomass, ethanol, acetic acid, and glycerol yields were expressed in g/g_glucose_, and calculated using Equation 1:


$$Y\,i\,e\,l\,d_{C\,o\,m\,p\,o\,u\,n\,d}=\frac{{\left[C\,o\,m\,p\,o\,u\,n\,d\right]}_{t\,12\,o\,r\,t\,15}-{\left[C\,o\,m\,p\,o\,u\,n\,d\right]}_{t\,0}}{{\left[G\,l\,u\,c\,o\,s\,e\right]}_{t\,0}-{\left[G\,l\,u\,c\,o\,s\,e\right]}_{t\,12\,o\,r\,t\,15}}\;\left(1\right)$$

Where *t*0 refers to the sample taken at 0 h, *t*12 at 12 h, and *t*15 at 15 h from the start of the screening. The specific growth rate was computed by calculating the linear regression of the natural logarithm of the OD_600_ value between 4 and 10 h (at least 4 time points).

#### Cultivation in the BioLector I

Yeast cells from a cryo-stock were inoculated the day prior the screening in 5 mL Delft medium and grown overnight at 30°C in 50-mL tubes. Cells were then inoculated in a suitable medium to a final volume of 200 μL using CELLSTAR black clear-bottom 96-well microtiter plates (Greiner bio-one) and sealed with AeraSeal films (Sigma-Aldrich). Initial OD_600_ was 0.2 for wells containing SWSHs and 0.1 for all other samples. The temperature was set to 30°C with 85% humidity, shaker frequency was 900 rpm, and cycle time was 30 min. Filter properties are described in section “BioLector I Filters and Analysis” and Supplementary Table 5. All cultivation conditions were investigated in triplicates.

#### Intracellular pH Calibration

Parental yeast strains and those bearing sfpHluorin were taken from a cryo-stock and grown overnight in Delft medium. In the morning, cells were re-inoculated at an OD_600_ of 0.4 in 100-mL baffled flasks containing 15 mL Delft medium and grown at 30°C and 200 rpm. A fresh 10 × digitonin stock solution (10 mg/mL in MilliQ-H_2_O) was prepared by mixing at 70°C until a clear solution was obtained. Upon reaching an OD_600_ of ∼1, 10 mL of culture was harvested and centrifuged for 3 min at 3,000 rpm. The cell pellet was washed once with phosphate-buffered saline (PBS) at pH 5, resuspended in 10 mL PBS (pH 7.4) containing 100 μg/mL digitonin, and incubated for 10 min at room temperature with shaking at low rpm. Cells were then centrifuged, washed once with PBS (pH 7.4), and resuspended in PBS (pH 7.4) to an OD_600_ of 20. Cells were added to citric acid/Na_2_HPO_4_ buffer, whose pH ranged from 4.5 to 8, to a final OD_600_ of 0.5 and in a final volume of 200 μL. Fluorescence was measured in a BioLector I using CELLSTAR black clear-bottom 96-well microtiter plates. Measurements were taken 30 min after the addition of cells. Fluorescence was plotted against pH and calibration curves were generated.

### BioLector I Filters and Analysis

The emission/excitation filters used in this study are summarized in Supplementary Table 5. At each time point, background fluorescence from the parental strain was subtracted from the fluorescence signal of strains bearing a biosensor. The signals from OxPro (ymYPET), GlyRNA (mTurquoise2), and RPL13A-mTurquoise2 (mTurquoise2) biosensors were normalized to the mCherry fluorescence of the pTEFmut8-mCherry construct. Instead, for QUEEN-2m and sfpHluorin, the ratio between the filters E-OP-341 and E-OP-304 was computed. In all cases, samples were analyzed in triplicates, and the mean and standard deviation among replicates were computed after calculating the ratio. When selecting the fluorescent proteins, we considered the following aspects: spectrum overlap, brightness, monomeric structure, and pKa < 5. Further details can be found in section 1.1 of Supplementary Material.

### Statistical Analysis

Pairwise comparisons were carried out in R (R Core Team, 2020), using unpaired Student’s *t*-test. Statistical significance was defined as follows: *^ns^p* > 0.05; *p ≤ 0.05; ^**^p ≤ 0.01, and ^***^p ≤ 0.001.

### Deposition to Addgene

Plasmids will be available from the Addgene repository^3^ using IDs (177705-177712) or by contacting the corresponding author.

## Results

### Five Biosensors Are Selected to Monitor the Yeast Intracellular Status During Stress

Five biosensors already proven to function in yeast and capable of detecting key intracellular parameters were selected from the literature (Figure 1A, Table 1, and Supplementary Table 5). They included ratiometric biosensors (pH and ATP concentration) and intensiometric biosensors (ribosome production, oxidative stress, and glycolytic flux), also selected on the need to match different fluorescent spectra if combined.

ATP is a crucial molecule in the energetic balance of the cell and exploring its fluctuations over time would reveal the energy fluxes associated with stress responses. Therefore, QUEEN-2m was selected as biosensor for this parameter (Yaginuma et al., 2014; Takaine et al., 2019). QUEEN-2m is biosensor based on a circularly permutated GFP, whose fluorescent intensity changes upon binding of ATP.

The intracellular pH biosensor sfpHluorin (Reifenrath and Boles, 2018) represents an improved version of the more commonly used pHluorin (Nygård et al., 2014). Owing to its greater pH stability, it is more suitable for industrial applications, as the elevated amount of weak acids in those substrates leads to acidification of the cytosol (Guldfeldt and Arneborg, 1998).

Furaldehydes and phenolic compounds in lignocellulosic biomass are often associated with redox imbalance because their detoxification requires NAD(P)H as a cofactor (Deparis et al., 2017; Liu, 2018). OxPro (Oxidative stress Probe) was selected as an oxidative stress sensor (Zhang et al., 2016). This biosensor is based on a synthetic promoter driving the expression of a fluorescent protein dependent by the activation of the transcription factor YAP1, the main oxidative stress mediator in yeast (Estruch, 2000). This construct formed part of a circuit capable of regenerating NADPH when the cell required it (Zhang et al., 2016).

In many fermentation processes, end products are synthesized starting from sugars (Francois et al., 2020; Maicas, 2020; Sharma et al., 2020). GlyRNA (Glycolytic RNA probe) was selected as an aptameric sensor for glycolytic flux, because degradation of its mRNA is sensible to the intracellular concentration of fructose-bisphosphate (Ortega et al., 2021). Given that the sensor’s response decreases with an increasing concentration of fructose-bisphosphate, a negative peak denotes maximum glycolytic flux.

Lastly, ribosomes have been suggested to control the lifespan of cells and might improve tolerance to growth inhibitors (Steffen et al., 2008; Gonskikh and Polacek, 2017). Therefore, to monitor ribosome production, RPL13A, one of the proteins in the 60S ribosomal subunit, was tagged with a fluorescent protein as done previously to correlate lifespan and ribosome levels (Janssens and Veenhoff, 2016). This biosensor, referred to as RibPro (Ribosome Probe), with the tagging of the endogenous RPL13A with a fluorescent protein, offered two important advantages. First, it avoided the need to introduce an additional tagged copy of the same gene, which might have led to unwanted overexpression. Second, it allowed a more accurate readout, as using the promoter activity of a ribosomal protein might have overlooked post-transcriptional regulation of the corresponding mRNA (Roy et al., 2020).

In the case of intensiometric biosensors GlyRNA, OxPro, and RibPro, a constitutively expressed fluorescent reporter (constructed in plasmid LT1_33_pTEFmut8-mCherry) was added to normalize the biosensor output; thus ensuring reliable readouts and minimizing the effect of population heterogeneity (see section 1.2 of the Supplementary Material). As both QUEEN-2m and sfpHluorin are ratiometric probes, they did not require this addition.

### The Highly Efficient X2 Integration Site Is Conserved in *Saccharomyces cerevisiae* Strains

**FIGURE 2:**
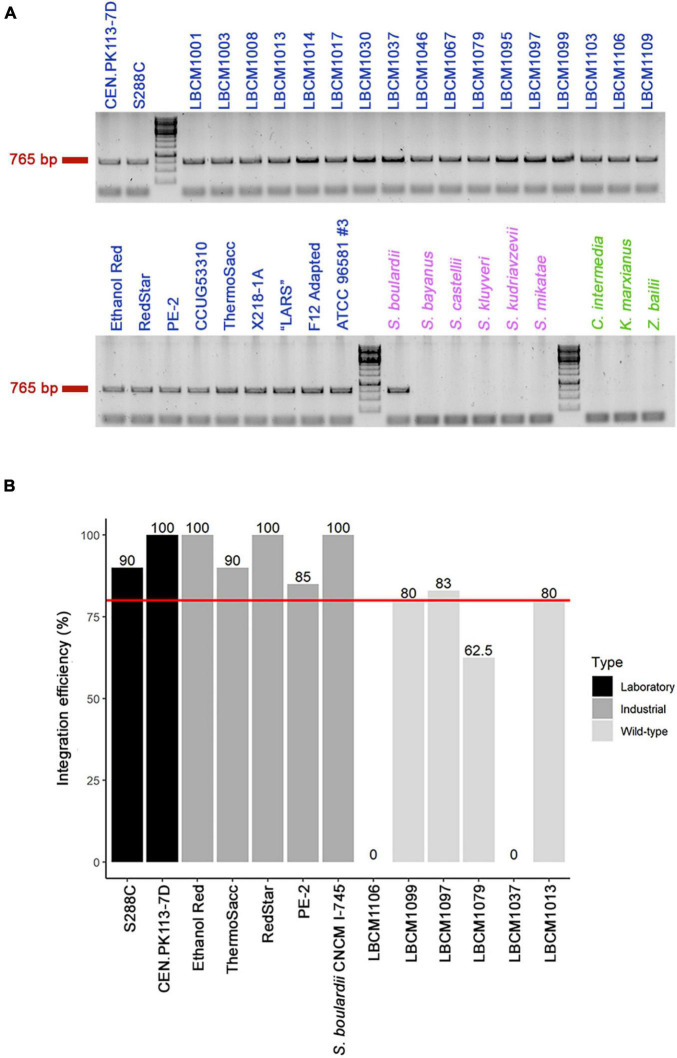
Presence of the X2 site and genome integration efficiency. **(A)** Presence of the conserved X2 site (band at 765 bp) was verified by colony PCR in multiple yeast strains, including 28 *Saccharomyces cerevisiae* (blue), six other *Saccharomyces* (violet), and three non-*Saccharomyces* (green) strains (see Table 2 for a full list of yeast strains). **(B)** Assessment of genome integration efficiency in 13 of the 29 *Saccharomyces* (28 *S. cerevisiae* and 1 *S. boulardii*) strains that harbored the X2 site was carried out using CRISPR-Cas9 genome editing technology. The red line represents 80% integration efficiency and the value for each strain is reported above the bar.

The proposed biosensor toolbox offers a simple and marker-free integration method that allows: (1) easy investigation of multiple yeast strains, (2) the possibility to avoid selection markers, and (3) stable expression of the fluorescent biosensors. In *S. cerevisiae*, the HO site is a common target for genome integration of a desired construct/pathway as it does not affect growth (Baganz et al., 1997). To allow use of the toolbox even in strains with HO-integrated constructs/pathways, we explored the possibility of integrating the biosensors in another site. Fourteen other safe-to-use sites offering elevated and stable expression have been described in *S. cerevisiae* (Mikkelsen et al., 2012). One of the most used ones is the X2 site on chromosome X. Owing to its location between two essential genes, GCD14 and CCT7, we hypothesized that this site could be conserved across yeast species and genera. Therefore, the presence of the X2 site was checked by colony PCR in laboratory, industrial, and wild-type *S. cerevisiae* strains, as well as in other *Saccharomyces* and non-*Saccharomyces* strains (Table 2). The X2 site was present in all 28 *S. cerevisiae* strains and *Saccharomyces boulardii* CNCM I-745, which shares 95% genome homology with *S. cerevisiae* (Khatri et al., 2017), but was absent from the remaining eight yeasts tested (Figure 2A). The sequence of the locus was blasted in NCBI to confirm that the missing band was not caused by primer mismatch. Given the above result and the dominant role of *S. cerevisiae* in bioindustry (Kampranis and Makris, 2012), this site seemed a good candidate for genome integration and for exploring the diversity within this genus.

CRISPR-Cas9 editing technology has been shown to improve genome integration efficiency (Akhmetov et al., 2018). Therefore, we used the construct LT1_33_pTEFmut8-mCherry to test genome integration efficiency in 13 of the 29 X2-positive strains. First, we designed a sgRNA (oligos LT58) targeting the region of interest and inserted it in a Cas9-expressing vector (final plasmid YN2_1_LT58_X2site, Addgene ID: 177705). Integration efficiency was > 80% for all laboratory and industrial strains tested, but between 60 and 80% for several Brazilian wild-type strains (Figure 2B). However, strains LBCM1037 and LBCM1106 showed no integration success even after repeating the transformation procedure 3 times. To determine the cause of such low efficiency, the X2 site was sequenced in the 13 strains harboring it (Supplementary Figure 1). The sequence for 12 of the 13 strains was very similar to that of CEN.PK113-7D; whereas the sequence of LBCM1106 was substantially different, including in the target sgRNA region. Therefore, optimization of the sgRNA sequence might be sufficient to improve integration efficiency. Prior any integration into a yeast strain not presented in this study, assessment of the presence of the X2 site should be performed. In addition, the presence of conserved single nucleotide polymorphisms and deletions in the three different yeast types (laboratory, industrial, and wild-type) pointed to possible similarities between strains (Supplementary Figure 1).

### Genome-Integrated Biosensors Do Not Affect the Central Metabolism of Yeast

To test the biosensors in the toolbox, we decided to continue the experiments using *S. cerevisiae* CEN.PK113-7D and Ethanol Red, as representatives of laboratory and industrial strains, respectively. All biosensors were first constructed as plasmids using the MoClo Modular Cloning System Plasmid Kit (Lee et al., 2015). No yeast selection markers were necessary as a dummy sequence containing STOP codons in different shift-frames was employed instead (oligos LT179_F and LT179_R). Then, after linearization, yeasts were transformed using CRISPR-Cas9 genome editing technology. Cells bearing the Cas9 plasmid were selected on medium containing G418, as it was assumed that they had most likely integrated the linearized donor DNA with the desired biosensor (see Supplementary Table 3 for a list of constructs). After selection of the correct clones by colony PCR, the Cas9 plasmid was cured to obtain the marker-free strains.

**FIGURE 3:**
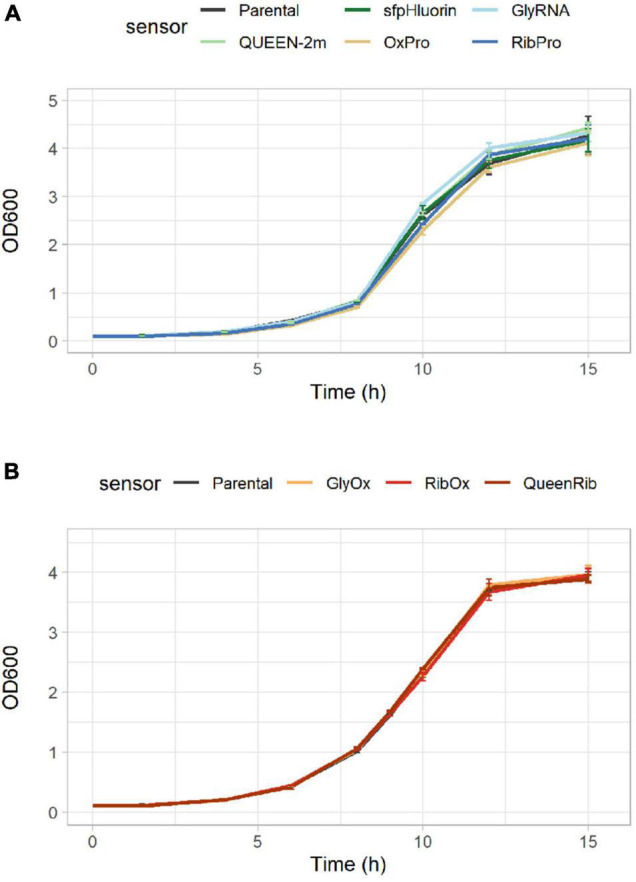
Growth comparison of CEN.PK113-7D strains bearing biosensors. Growth curves showing the increase in optical density at 600 nm (OD600) over time for the parental CEN.PK113-7D strain and its derivatives bearing either **(A)** one or **(B)** a pair of biosensors.

As the selected biosensors would monitor the intracellular environment, their presence should not cause any significant alteration in cellular metabolism. CEN.PK113-7D strains bearing single biosensors were cultured anaerobically in 500-mL flasks in Delft medium (minimal defined synthetic medium) to quantify their growth performance. Neither the growth curves (Figure 3A) nor the maximum specific growth rates (Table 4) of strains bearing single biosensors showed any significant difference with respect to the parental strain. The same trend was observed also for the yields of key metabolites at the beginning of incubation and during stationary phase; only the acetic acid yield of the strain carrying sfpHluorin differed from that in the parental strain (Table 4).

**TABLE 4:** Physiological rates and yields of yeast strains harboring singular biosensors.

CEN.PK113-7D strain	Specific growth rate (h^–1^)	Biomass yield (g/g)		Ethanol yield (g/g)		Glycerol yield (g/g)		Acetic acid yield (g/g)	
		12 h	15 h	12 h	15 h	12 h	15 h	12 h	15 h
Parental	0.379 ± 0.000	0.090 ± 0.015	0.088 ± 0.008	0.388 ± 0.008	0.387 ± 0.006	0.167 ± 0.027	0.167 ± 0.03	0.018 ± 0.002	0.019 ± 0.000
QUEEN-2m	0.382 ± 0.002^ns^	0.098 ± 0.006^ns^	0.083 ± 0.004^ns^	0.393 ± 0.011^ns^	0.38 ± 0.009^ns^	0.150 ± 0.020^ns^	0.149 ± 0.019^ns^	0.019 ± 0.001^ns^	0.020 ± 0.001^ns^
sfpHluorin	0.372 ± 0.014^ns^	0.102 ± 0.005^ns^	0.091 ± 0.006^ns^	0.384 ± 0.012^ns^	0.374 ± 0.011^ns^	0.157 ± 0.026^ns^	0.157 ± 0.027^ns^	0.017 ± 0.001^ns^	0.018 ± 0.000*
GlyRNA	0.379 ± 0.009^ns^	0.093 ± 0.008^ns^	0.085 ± 0.002^ns^	0.392 ± 0.013^ns^	0.380 ± 0.009^ns^	0.163 ± 0.015^ns^	0.162 ± 0.021^ns^	0.016 ± 0.001^ns^	0.020 ± 0.001^ns^
OxPro	0.360 ± 0.033^ns^	0.088 ± 0.003^ns^	0.087 ± 0.006^ns^	0.389 ± 0.012^ns^	0.378 ± 0.009^ns^	0.147 ± 0.021^ns^	0.144 ± 0.022^ns^	0.018 ± 0.001^ns^	0.019 ± 0.001^ns^
RibPro	0.375 ± 0.011^ns^	0.094 ± 0.007^ns^	0.087 ± 0.004^ns^	0.39 ± 0.01^ns^	0.380 ± 0.012^ns^	0.148 ± 0.025^ns^	0.147 ± 0.024^ns^	0.017 ± 0.001^ns^	0.019 ± 0.001^ns^

^ns^p > 0.05; *p ≤ 0.05.

To allow detection of several intracellular parameters simultaneously, multiple biosensors were integrated in the same cell. Again, neither the growth curves (Figure 3B) nor the performance parameters of strains bearing multiple biosensors (GlyOx, RibOx, and QueenRib) differed from those of the parental CEN.PK113-7D strain (Table 5). Overall, the selected biosensors did not affect yeast growth and could reliably convey the cells’ physiological state during different growth conditions.

**TABLE 5:** Physiological rates and yields of yeast strains harboring multiple biosensors.

CEN.PK113-7D Strain	Specific growth rate (h^–1^)	Biomass yield (g/g)		Ethanol yield (g/g)		Glycerol yield (g/g)		Acetic acid yield (g/g)	
		12 h	15 h	12 h	15 h	12 h	15 h	12 h	15 h
Parental	0.418 ± 0.006	0.121 ± 0.006	0.125 ± 0.002	0.387 ± 0.002	0.376 ± 0.003	0.056 ± 0.002	0.055 ± 0.002	0.011 ± 0.000	0.015 ± 0.001
GlyOx	0.414 ± 0.005^ns^	0.120 ± 0.004^ns^	0.125 ± 0.003^ns^	0.387 ± 0.001^ns^	0.372 ± 0.002^ns^	0.054 ± 0.001^ns^	0.053 ± 0.001^ns^	0.011 ± 0.000^ns^	0.016 ± 0.000^ns^
RibOx	0.405 ± 0.003^ns^	0.123 ± 0.004^ns^	0.127 ± 0.003^ns^	0.390 ± 0.000^ns^	0.375 ± 0.002^ns^	0.052 ± 0.001^ns^	0.052 ± 0.001^ns^	0.011 ± 0.000^ns^	0.016 ± 0.001^ns^
QueenRib	0.420 ± 0.011^ns^	0.116 ± 0.002^ns^	0.126 ± 0.002^ns^	0.387 ± 0.001^ns^	0.374 ± 0.002^ns^	0.056 ± 0.002^ns^	0.056 ± 0.002^ns^	0.011 ± 0.000^ns^	0.016 ± 0.001^ns^

*^ns^p > 0.05.*

### Combining Biosensors in Different Carbon Sources Gives a Reliable Fluorescence Output

**FIGURE 4:**
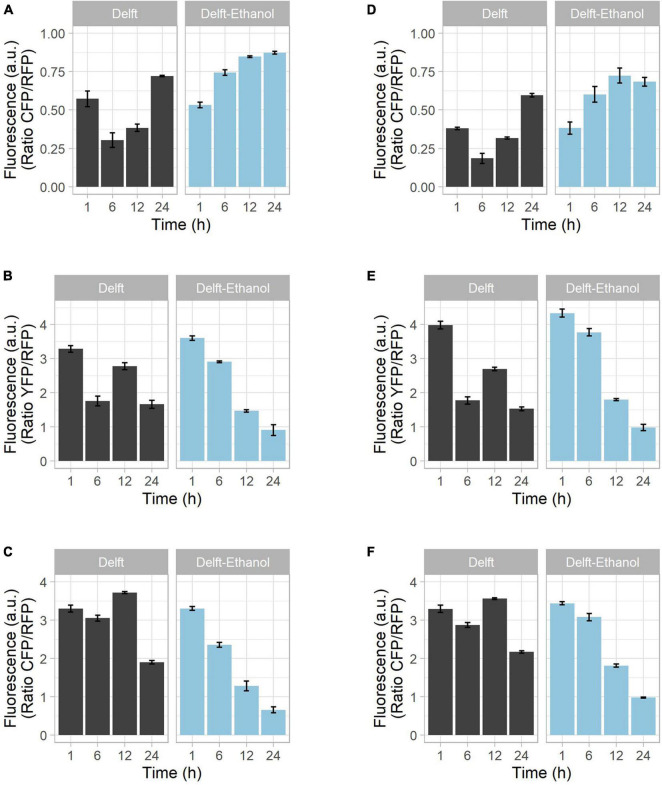
Growth of strains on different carbon sources and biosensor combination. CEN.PK113-7D strains bearing biosensors were grown in synthetic minimal Delft medium containing either 20 g/L glucose (black bars) or 20 g/L ethanol (blue bars) as sole carbon source. Mutants with **(A–C)** a single biosensor or **(D–F)** pairs of biosensors were tested to determine the reliability of the fluorescence output. The tested intracellular parameters included **(A,D)** glycolytic flux (via GlyRNA and GlyOx), **(B,E)** oxidative stress (via OxPro and GlyOX), and **(C,F)** ribosome production (via RibPro and RibOx). Using glucose as carbon source, the time points denote lag phase (1 h), exponential phase (6 h), diauxic shift (12 h), and stationary phase (24 h). With growth on ethanol, time points denote lag phase (1–12 h) and active growth (24 h).

Simultaneous detection of multiple intracellular parameters could point to correlations among them. To this end, we combined pairs of biosensors with non-overlapping spectral properties in the same cell and assessed their fluorescent signal. As in the case of singular biosensors, paired biosensors were all introduced into the X2 site, together with the normalization construct. The GlyRNA-OxPro (GlyOx) combination allowed simultaneous detection of oxidative stress and glycolytic flux (LT2_14_GlyOx as plasmid for integration, Supplementary Table 3); whereas the RibPro-OxPro (RibOx) pair allowed simultaneous detection of ribosome production and oxidative stress (RPL13A-mTuquoise2 Tag plus LT2_7_OxPro as plasmid for integration, Supplementary Table 3). The cells carrying either single or paired biosensors were grown on two distinct carbon sources, glucose and ethanol, to determine if different metabolic pathways affected the fluorescent response (Figure 4 and Supplementary Figure 2). Under aerobic batch conditions, *S. cerevisiae* consumes all the glucose through glycolysis and fermentation, converting it into ethanol. Once glucose is exhausted, ethanol can be used as an alternative carbon source through the tricarboxylic acid cycle (Pfeiffer and Morley, 2014). If ethanol is used as the sole carbon source, only the tricarboxylic acid cycle is activated.

During growth on glucose, the signal from the GlyRNA biosensor was low in exponential phase, but increased as the glucose was exhausted (Figure 4A and Supplementary Figures 2A,B). In contrast, when grown solely on ethanol, the GlyRNA signal increased from the start, indicating no active glycolytic flux (Figure 4A and Supplementary Figures 2A,B). Oxidative stress fluctuated over time in glucose-grown cells, with a minor increase of OxPro biosensor fluorescence during the diauxic shift (Figure 4B and Supplementary Figures 2C,D); whereas ethanol resulted in a continuous decrease (Figure 4B and Supplementary Figures 2C,D). The same trend was observed for ribosome production (Figure 4C and Supplementary Figures 2E,F). The bump in RibPro fluorescence detected during the diauxic shift can be explained by the switch from a fermentative to a respiratory metabolism, which coincides with more protein synthesis. For the same reason, activation of the tricarboxylic acid cycle during the diauxic shift coincides with an increased release of reactive oxygen species (Balaban et al., 2005), which yeast cells need to acclimate to. In the case of ethanol-grown cells, this adaptation is missing, and the trends are comparable with the second growth on ethanol of glucose-grown cells.

Overall, we showed that the fluorescent outputs from both single (Figures 4A–C) and combined (Figures 4D–F) biosensors displayed comparable trends (Supplementary Figure 2). Considering that the use of these biosensors should be qualitative and for the comparison of trends, we confirm a reliable readout from the strains with combined biosensors.

### Tight Regulation Between ATP Production and Intracellular pH

**FIGURE 5:**
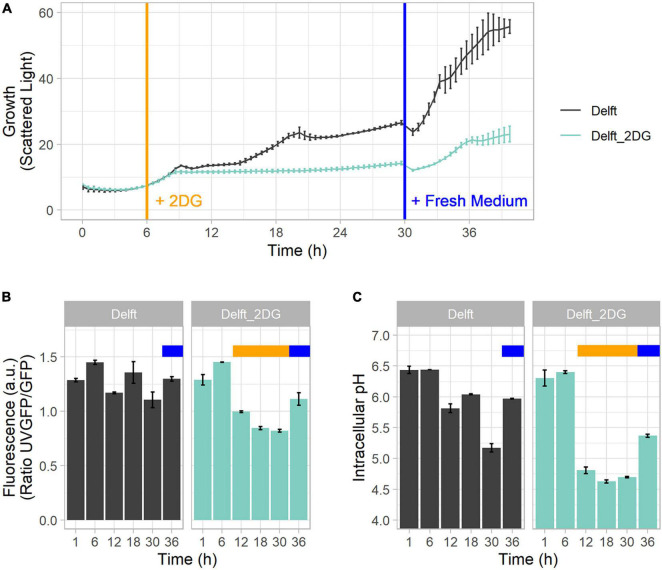
Mutual connection between ATP and intracellular pH. **(A)** Growth curve profile of CEN.PK113-7D in Delft medium and Delft medium spiked at 6 h (orange line) with 2-deoxy-D-glucose (2DG) to stop growth and ATP production by glycolysis. In both conditions, medium was replaced at 30 h with fresh Delft medium (blue line). **(B)** ATP concentration detected with sfpHluorin. **(C)** Intracellular pH detected with QUEEN-2m. In Delft medium, the time points denote lag phase (1 h), first exponential phase (6 h), diauxic shift (12 h), second growth on ethanol (18 h), stationary phase (30 h), and growth after additional glucose supplementation (36 h). The orange bar denotes the presence of 2DG in the medium; the blue bar denotes replacement with fresh medium.

ATP production and intracellular pH are two key indicators of the state and activity of cells. To study the correlation between them, CEN.PK113-7D strains bearing sfpHluorin and QUEEN-2m were cultured in Delft medium and spiked or not with 2-deoxy-D-glucose (2DG, final concentration of 0.5 g/L) at 6 h to inhibit the glycolytic flux, ATP production, and exponential growth (Figure 5A) (Ortega et al., 2020). At 30 h, the medium in spiked and non-spiked cultures was replaced with fresh Delft medium (Figure 5A). The QUEEN-2m biosensor revealed that ATP was produced when cells grew exponentially on either glucose (6 h) or ethanol (18 h) (Figures 5A,B). At the same time, the sfpHluorin biosensor revealed that intracellular pH was maintained between 6 and 6.5 during growth, but dropped below 6 when ATP was no longer produced (12 and 30 h) (Figure 5C). Eventually, if glucose was added during stationary phase (36 h), new ATP was produced and pH stabilized around 6 again (Figures 5B,C).

When 2-deoxy-D-glucose was added to the medium at 6 h, ATP production decreased, and intracellular pH dropped below 5 (time points 12–30 h) (Figures 5B,C). Upon medium replacement at 30 h, both ATP production and intracellular pH were restored (Figures 5B,C). This finding highlights the link between intracellular pH and ATP production. Intracellular pH regulation needs high amounts of ATP to power membrane pumps. Hence, in the absence or limitation of ATP, pH regulation cannot function properly and intracellular acidification ensues, as confirmed by a decrease in cytoplasmic pH following inactivation of an ATP-driven proton pump (Isom et al., 2018).

### Activation of the Oxidative Stress Response and Correlation With Intracellular pH in the Presence of Stressors

**FIGURE 6:**
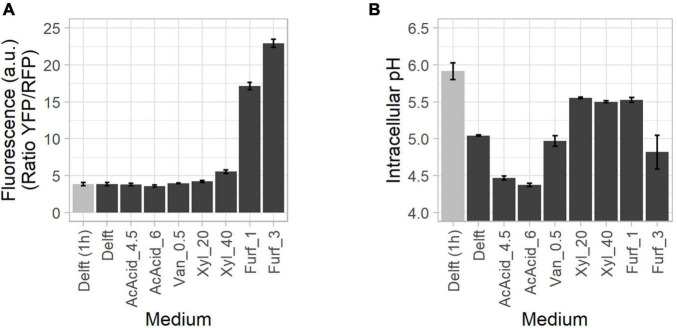
Oxidative stress activation and drop in intracellular pH during growth in the presence of lignocellulosic inhibitors. CEN.PK113-7D cells were grown in the presence of lignocellulose-derived stressors, including acetic acid (AcAcid) at 4.5 and 6 g/L, vanillin (Van), xylose (Xyl) at 20 and 40 g/L, and furfural (Furf) at 1 and 3 g/L. **(A)** Oxidative stress response activation (highest values, in black) assessed with the OxPro biosensor. **(B)** Intracellular pH (lowest values, in black) assessed with the sfpHluorin biosensor. “Delft (1 h)” (in gray) refers to the parameter at 1 h after the onset of screening.

Oxidative stress is very common in industrial bioethanol production plants due to the elevated amount of furaldehydes and phenolics released from the degradation of sugars and lignin (Sjulander and Kikas, 2020). Moreover, substrate pre-treatments release also weak acids (affecting the intracellular pH) and sugars (Deparis et al., 2017). To investigate changes in intracellular pH (using sfpHluorin) and activation of the oxidative stress response (using OxPro) under industrial-type settings, cells were subjected to four individual stressors commonly found in lignocellulosic hydrolysates, namely acids, phenolics, furaldehydes, and sugars (Figure 6 and Supplementary Figures 3, 4).

Acetic acid is a common and abundant weak acid in lignocellulosic hydrolysates, where it is generally found in its protonated form (Cunha et al., 2019). Upon diffusing into the cell, it releases a proton, acidifying the cytosol (Ullah et al., 2012). When cultured in acetic acid at 4.5 and 6 g/L, the cells did not show any activation of the stress response (Figure 6A and Supplementary Figures 3A, 4A). Intracellular pH remained stable until the end of exponential phase (Supplementary Figures 3B, 4B), but dropped thereafter more than with any of the other stressors tested (Figure 6B), probably due to an ATP shortage (see section “Tight Regulation Between ATP Production and Intracellular pH”). Note that acetic acid (pKa = 4.75) increase its inhibitory effect on growth as the pH in the medium (Pampulha and Loureiro-Dias, 1989).

Furfural and vanillin are known for causing a redox imbalance due to NAD(P)H depletion (Liu, 2018). Indeed, furfural elicited a strong oxidative stress response (Figure 6A), especially in lag phase (Supplementary Figures 3C, 4C). The timing of the response can be explained by the need for yeast cells to detoxify the medium prior to starting exponential growth (Liu, 2018). In contrast, vanillin did not activate the oxidative stress response (Figure 6A) and intracellular pH remained constant (pH 5) during the entire period (Supplementary Figures 3D, 4D).

Finally, xylose, which cannot be metabolized by any of the two strains, was used to induce osmotic stress, a common event with lignocellulosic substrates (Deparis et al., 2017). The oxidative stress response showed moderate activation during exponential growth (Figure 6A and Supplementary Figures 3E, 4E) while intracellular pH was stable over the growth period (Figure 6B and Supplementary Figures 3F, 4F).

### Capturing and Correlating Physiological Responses in an Industrial Setup

To demonstrate application of the toolbox under typically harsh industrial settings (Deparis et al., 2017), the biosensor-containing strains were tested in synthetic wheat straw hydrolysate (SWSH). Wheat straw is an abundant residue of low commercial value (Talebnia et al., 2010). SWSH was tested in aerobic conditions at 50 and 80% of total compound concentration (Table 3) to assess dose-dependent effects. Both laboratory (CEN.PK113-7D) and industrial (Ethanol Red) *S. cerevisiae* were used to highlight differences and similarities in the stress response. SWSH was expected to cause elevated oxidative stress and pronounced redox imbalance due its high furfural, 5-(hydroxymethyl) furfural, and vanillin content (Liu, 2018). At the same time, its production of fermentable (e.g., glucose, mannose, and galactose) and non-fermentable (e.g., xylose and arabinose) sugars was expected to cause moderate osmotic stress (Wang et al., 2013).

**FIGURE 7:**
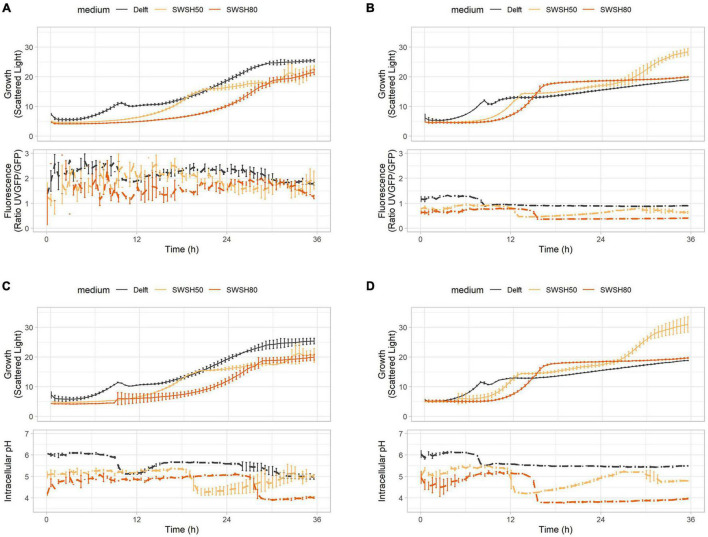
ATP production and intracellular pH during growth in synthetic wheat-straw hydrolysate (SWSH). **(A,B)** Growth curves and ATP concentration for **(A)** CEN.PK113-7D and **(B)** Ethanol Red strains harboring the QUEEN-2m biosensor. **(C,D)** Growth curves and intracellular pH for **(C)** CEN.PK113-7D and **(D)** Ethanol Red strains harboring the sfpHluorin biosensor. Cells were grown in Delft medium (black line), SWSH diluted at 50% with Delft medium (SWSH50; orange line), and SWSH diluted at 80% with Delft medium (SWSH80; red line).

**FIGURE 8:**
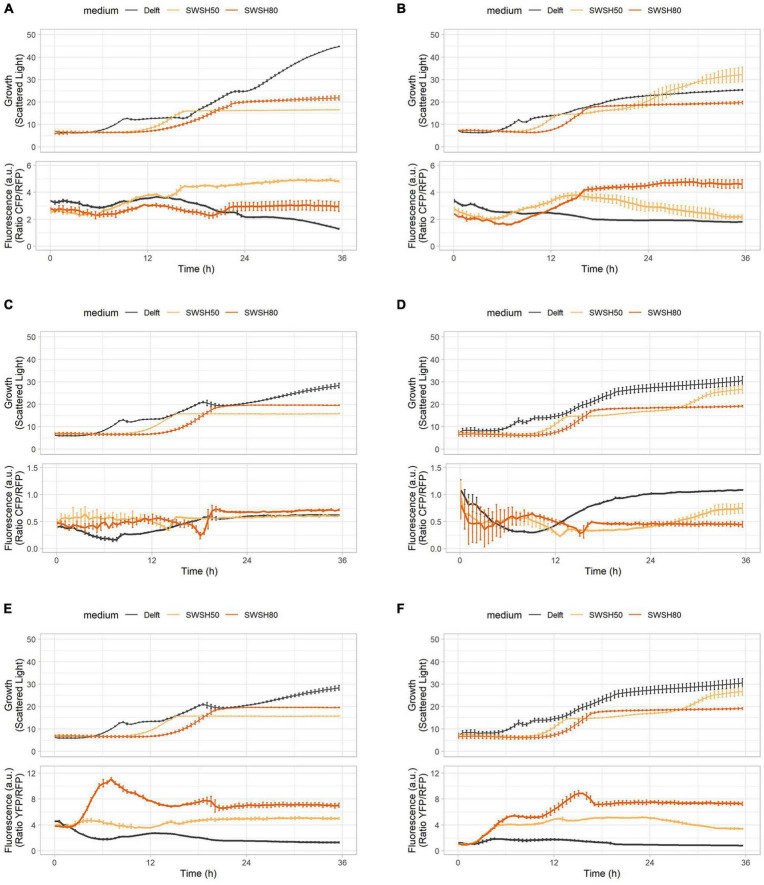
Oxidative stress, ribosome production, and glycolytic flux during growth in synthetic wheat-straw hydrolysate (SWSH). **(A,B)** Growth curves and ribosome production for **(A)** CEN.PK113-7D and **(B)** Ethanol Red strains harboring the RibOx biosensor. **(C,D)** Growth curves and glycolytic flux for **(C)** CEN.PK113-7D and **(D)** Ethanol Red strains harboring the GlyOx biosensor. **(E,F)** Growth curves and oxidative stress for **(E)** CEN.PK113-7D and **(F)** Ethanol Red strains harboring the GlyOx biosensor. Cells were grown in Delft medium (black line), SWSH diluted at 50% with Delft medium (SWSH50; orange line), and SWSH diluted at 80% with Delft medium (SWSH80; red line).

As highlighted in previous experiments, intracellular pH and ATP were strictly interconnected in both CEN.PK113-7D and Ethanol Red cells (Figure 7). Intracellular pH remained stable if ATP was produced from hexoses or ethanol (Figures 7A,B), but its baseline was seen to decrease with increasing harshness of the medium (Figures 7C,D). Ribosome production (detected with RibOx) remained generally stable in CEN.PK113-7D in both Delft medium and different types of SWSH (Figure 8A). Instead, ribosomes of Ethanol Red cells grown in SWSHs became more abundant during exponential phase, peaked at the diauxic shift, and started to decline thereafter (Figure 8B). Even though SWSH50 and SWSH80 were similar, ribosomes remained more numerous in cells grown on SWSH80, probably due to a prolonged diauxic shift, which extended beyond 36 h. The glycolytic flux (detected with GlyOx) in CEN.PK113-7D rose sharply during exponential phase but came to a halt at the beginning of the diauxic shift (Figure 8C). In Ethanol Red, the glycolytic flux rose slowly during exponential phase, started declining during the diauxic shift, and stabilized in stationary phase (note that in SWSH80, the diauxic shift was not over at 36 h) (Figure 8D). Lastly, the oxidative stress pattern (monitored with GlyOx) differed between the two strains. In CEN.PK113-7D grown in SWSH80, oxidative stress peaked first in lag phase, then decreased, peaked again upon diauxic shift, and stabilized in stationary phase (Figure 8E). Oxidative stress in Ethanol Red, instead, kept increasing over time, peaked in exponential phase, and stabilized thereafter at a higher level with respect to the control condition (Figure 8F). These differences seen between the two strains might rely on the fact that being an industrial strain, Ethanol Red’s stress response is more adapted to face industrial conditions, while laboratory CEN.PK113-7D is not.

## Discussion

Bioindustries are constantly on the lookout for more robust and efficient microbial strains. On-line monitoring of physicochemical parameters in bioreactors (e.g., gas exit, pH, oxygen levels, and pressure) and analytical methods for quantifying various compounds already allow for performance estimations. However, tools capable of providing real-time information about the cell physiological or metabolic status are still inadequate. Their development would offer new insights on yeast physiology, thereby improving cell factories and providing new means for monitoring and controlling such processes. To this end, biosensors capable of detecting changing parameters in real time, represent a key resource (Alvarez-Gonzalez and Dixon, 2019).

In this study, we combined five genetically encoded fluorescent biosensors, monitoring intracellular ATP, pH, oxidative stress, ribosome production, and glycolytic flux, into a toolbox. All constructs were created using the MoClo Modular Cloning System Plasmid Kit (Lee et al., 2015), which allows for easy implementation of new biosensors (i.e., sensing other parameters) and adjustment of already existing ones (e.g., replacement of fluorescent proteins). A safe-to-use site situated on chromosome X was chosen for genome integration (Mikkelsen et al., 2012), which appeared to be conserved in all tested *S. cerevisiae* strains plus a commercial *S. boulardii* strain (Figure 2). For integration, an efficient and marker-free workflow using CRISPR-Cas9 genome editing technology was designed. Such an approach will favor the exploration of natural diversity in *S. cerevisiae* strains collected from nature, whose features might be of interest to the industrial sector. Indeed, the genetic and phenotypic variation in industrial strains has been shown to be low and the implementation of new improved genetic stocks might be the next step for bioindustries (Molinet and Cubillos, 2020). Moreover, we proved that the presence of these biosensors inside the cell did not affect the specific growth rate and yields of key intracellular metabolites (Tables 4, 5), allowing to use the system for quantitative and real-time monitoring.

By combining distinct biosensors in the same cell, we investigated possible correlations between different intracellular parameters. For example, we showed a tight connection between ATP concentration and intracellular pH, as well as followed simultaneously the oxidative stress response and variations in intracellular pH of cells grown in the presence of lignocellulose-specific stressors. Using SWSH-containing medium to mimic industrial conditions, we highlighted how the laboratory CEN.PK113-7D and industrial Ethanol Red *S. cerevisiae* strains responded differently to oxidative stress. In the laboratory strain, the response peaked during lag phase and then decreased over time; whereas in the industrial strain, the peak was seen during exponential growth. Therefore, this toolbox proves to be an instrument to study strain-specific and time-resolved differences in physiological responses. Implementing such multi-sensing tool together with high-throughput technology enables the parallel investigation of the intracellular status in known or newly identified candidate microorganisms. This approach would guide strain characterization ahead of more detailed high-resolution omics studies.

Some limitations of the toolbox remain unsolved. One is the impossibility to combine more than two biosensors in the same cell due to emission/excitation spectral overlap. If single-cell analysis such as flow cytometry is performed, one solution would be the co-culture of strains harboring different biosensor combinations to create a fluorescent footprint. For example, by co-culturing strains carrying either sfpHluorin, RibPro or GlyOx, it is possible to first filter mCherry^–^ cells (sfpHluorin) from mCherry^+^ cells (GlyOx and RibPro). Then, among the mCherry^+^ subpopulation, ymYPET^–^ cells (RibPro) can be separated from ymYPET^+^ cells (GlyOx). This strategy would enable the simultaneous detection of multiple intracellular parameters in the same bioreactor using flow-cytometry. Importantly, as the performance of different strains is not affected by the biosensor, co-culture does not lead to any bias. Real-time monitoring is attractive, but although some high-throughput instruments (such as the BioLector I) allow for detection of growth and fluorescence in real time, such measurements are not implemented in industrial bioreactors. Sampling of cells during the process is still possible, but additional fluorescence-detecting instrumentation, such as a fluorescent microscope or a flow cytometer, as well as subsequent analysis are necessary. Although this problem affects laboratory-scale yeast studies only in a limited way, it can be a stumbling block for industrial applications, where on-line measurements are common for process monitoring and control.

Although only examples and applications of the toolbox in population studies were presented, normalization to an additional fluorescent protein (see section 1.2 of the Supplementary Material) enables better single-cell analysis, an area of increasing interest in research and application. For instance, this toolbox could shed a light on unresolved and peculiar phenomena happening in industrial processes, such as the drop in productivity during certain high-gravity fermentations (Koppram et al., 2012) or near-zero growth (Boender et al., 2011; Vos et al., 2016). Moreover, the physicochemical gradients formed during large-volume fermentation processes cause the emergence of subpopulations within the cultures (Wehrs et al., 2019). Even within the same bulk population, different cells might behave differently and possess different characteristics (Heins and Weuster-Botz, 2018). These phenomena are referred to as population heterogeneity (Heins and Weuster-Botz, 2018). For example, flow cytometry studies have highlighted how cells at different growth stages and exposed to a varying external pH, have different intracellular pH and form subpopulations (Valli et al., 2005). Another single-cell study unveiled the increased tolerance to lignocellulosic inhibitors of cell populations harvested in early-stationary phase (Narayanan et al., 2017). Bioreactor subpopulations have been studied thanks to the use of propidium iodide and a fluorescent reporter expressed under the promoter of the ribosomal gene RPL22A, whose transcription has shown to be correlated with cell growth (Carlquist et al., 2012; Delvigne et al., 2015). Microfluidic devices and product biosensors revealed different production phases and subpopulations during *L*-valine generation in bacteria (Mustafi et al., 2014). The study focused on the subpopulations arising during bioprocesses can be the key to improve consistency of existing bioprocesses, understand and implement new robustness features, and direct the next generation of cell factories.

The here presented toolbox offers two main opportunities. First, it allows for the investigation and the acquisition of a deeper knowledge on the intracellular state of cells during bioprocesses. Second, it has the potential to be a tool for monitor of industrial bioproduction processes, especially when coupled with biosensors able to detect the desired product (Marsafari et al., 2020; Zhang and Shi, 2021). The easy implementations into the toolbox of newly developed or already-existing biosensors able to sense additional intracellular parameters of interest would further promote the comprehension of microbial behaviors in such processes. For instance, a recently developed biosensor able to detect the unfolded protein response might be useful in the development of the new generation of yeast expressing heterologous proteins (Peng et al., 2021). The design of the YAP1-based biosensor for oxidative stress has been improved and showed potential applications also in the probiotic yeast *S. boulardii* (Dacquay and McMillen, 2021). Acid stress resulting from acids in the substrates and/or products used in bioindustries can be sensed with HAA1-based or acid-responsive-promoter-based biosensors and used for screening new acetic-acid-producing strains (Hahne et al., 2021; Mormino et al., 2021). New condition-specific biosensors can be developed thanks to the use of yeast native promoters (Xiong et al., 2018). Therefore, future studies using biosensors should focus both on the single-cell aspect and on the performance comparison of industrially relevant and newly isolated strains in different substrates and conditions to point out robustness features.

## Data Availability Statement

The datasets presented in this study can be found in online repositories. The names of the repository/repositories and accession number(s) can be found below: https://www.addgene.org, 177705–177712.

## Author Contributions

LT performed the experiments and wrote the manuscript. LO and PR supervised and contributed for the discussion of the collected data. All authors conceived the study and contributed for the correction of the manuscript prior submission.

## Conflict of Interest

PR has financial interests in Enduro Genetics ApS. The remaining authors declare that the research was conducted in the absence of any commercial or financial relationships that could be construed as a potential conflict of interest.

## Publisher’s Note

All claims expressed in this article are solely those of the authors and do not necessarily represent those of their affiliated organizations, or those of the publisher, the editors and the reviewers. Any product that may be evaluated in this article, or claim that may be made by its manufacturer, is not guaranteed or endorsed by the publisher.

## Footnotes

1
www.twistbioscience.com

2
https://dna.macrogen-europe.com

3
https://www.addgene.org
