# Supplementary material for: Real-Time Monitoring of the Yeast Intracellular State During Bioprocesses With a Toolbox of Biosensors
Source: Front Microbiol. 2022 Jan 7;12:802169. doi: 10.3389/fmicb.2021.802169 (PMC8776715; doi:10.3389/fmicb.2021.802169)
Supplement: Supplementary file 1 [file Data_Sheet_1.PDF]

Supplementary Material

1. Supplementary Data

1.1. Selection of fluorescent proteins

The sensors for oxidative stress, glycolytic flux, and ribosome abundance employed in this study were based on promoter activation, mRNA degradation, and protein tagging, respectively. This allowed the fluorescent protein to be customised according to the needs of each experiment. In this study, monomeric (to avoid protein aggregates) and bright (for easy detection) fluorescent proteins with maturation times < 40 min (for fast detection of intracellular events) were selected; they included mTurquoise2, ymYPET, and mCherry (Botman et al., 2019; Lambert, 2019). Another crucial determinant for an optimal signal was the pKa of the fluorescent proteins (< 5 in the three selected proteins). Although the intracellular pH is expected to be between 5.9 and 6.4 throughout brewery fermentation (Rowe et al., 1994), it can drop to 4.5 when using weak acids in the medium (Guldfeldt and Arneborg, 1998) or even 4 during glucose deprivation (Elsutohy et al., 2017). As both weak acids release and glucose depletion are likely to occur when yeast grows on lignocellulosic substrates, a pKa < 5 was an essential prerequisite in the present study.

1.2. Fluorescence normalisation method

Biosensors can be either ratiometric or intensiometric. In ratiometric probes, multiple (generally two) excitation/emission spectra are recorded to detect a desired feature (e.g. changes in pH, ions, and ATP). In this case, normalisation is performed when computing the ratio and any differences in the number of biosensors within a cell population are removed. For example, pH sensors are generally excited with two wavelengths and then assessed in one emission spectrum that changes depending on the pH; therefore, the resulting ratio varies according to pH as well.

Intensiometric probes increase/decrease in intensity based on the response to a changing condition. There are different ways to normalise the fluorescent signal derived from an intensiometric fluorescent biosensor. At the population level, normalisation might rely on optical density at 600 nm (OD_{600}) and the fluorescent signal is divided by the OD_{600} value. At the single-cell level, the averaged signal emitted by each cell might be used for normalisation. Alternatively, the signal from the biosensor can be normalised to the fluorescence coming from an additional constitutively expressed fluorescent protein (as in the case of the intensiometric biosensors in this study). Generally, when the sensor is introduced in the cell on a plasmid, normalisation by another fluorescent protein (in the same plasmid) is used to account for potential differences in the number of plasmids inside each cell within a population. From a single-cell point of view, this approach would correct for the signal coming from a cell with three copies of the sensor plasmid being most likely higher than that coming from a cell with only one copy, even though more copies do not necessarily imply greater biosensor activity. This normalisation method is crucial also for genome-integrated biosensors (as in this study). For instance, cells in mid-exponential phase are more metabolically active than those in stationary phase. Moreover, at the single-cell level, it might happen that two cells are in the same growth phase and possess the same number of biosensor copies (as it is genome-integrated), but might have different metabolic activities due to population heterogeneity, and therefore different fluorescent intensities coming from the biosensor. Therefore, normalisation to a constitutively expressed protein (pTEFmut8-mCherry) was the preferred method in all intensiometric biosensors employed in this study. The pTEFmut8 promoter was chosen as it had 70% of the native

promoter activity and resulted in constant expression throughout the experiment (Nevoigt et al., 2006).

2. Supplementary Figures and Tables

2.1. Supplementary Tables

Supplementary Table 1. Trace metals and vitamin solutions. Composition of (A) trace metal and (B) vitamin solutions used in Delft medium.

A. Trace metal solution 1000×		
EDTA	15	g/L
$ZnSO_4 \cdot 7\ H_2O$	4.5	g/L
$MnCl_2 \cdot 4\ H_2O$	0.8	g/L
$CoCl_2 \cdot 6\ H_2O$	0.3	g/L
$CuSO_4 \cdot 5\ H_2O$	0.3	g/L
$Na_2MoO_4 \cdot 2\ H_2O$	0.4	g/L
$CaCl_2 \cdot 2H_2O$	4.5	g/L
$FeSO_4 \cdot 7H_2O$	3	g/L
H_3BO_3	1	g/L
KI	0.1	g/L

B. Vitamin solution 1000×		
d-Biotin	0.05	g/L
Calcium D-(+)-pantothenate	1	g/L
Nicotinic acid	1	g/L
Myo-inositol	25	g/L
Thiamine HCl	1	g/L
Pyridoxine HCl	1	g/L
Para-aminobenzoic acid	0.2	g/L

Supplementary Table 2. Characterisation of the constructs used in the generation of plasmids for genome integration. List of DNA sequences adapted for the MoClo Modular Cloning System Plasmid Kit and added to it as level 0 plasmids. Some sequences were ordered from Twist Bioscience (*), while others were amplified from plasmids or oligos (*italics*).

Construct	Description	Sequence REF
*QUEEN-2m	Coding sequence of the QUEEN-2m biosensor used for sensing intracellular ATP concentrations. Adapted for the MoClo kit as type 3.	Addgene plasmid #129350; http://n2t.net/addgene:129350;
*sfpHluorin	Coding sequence of the sfpHluorin biosensor used for sensing intracellular pH. Adapted for the MoClo kit as type 3.	Addgene plasmid #115697; http://n2t.net/addgene:115697;
*ymYPET	Coding sequence of the yeast-optimised yellow fluorescent protein YPET. Adapted for the MoClo kit as type 3.	Addgene plasmid #118455; http://n2t.net/addgene:118455;
pTRX2_5xUAS	Sequence of the promoter pTRX2_5xUAS in the OxPro biosensor used for sensing oxidative stress. Adapted for the MoClo kit as type 2. Amplified from Addgene plasmid #124708 with oligos LT57.	Addgene plasmid #124708; http://n2t.net/addgene:124708;
pTEFmut8	Sequence of the promoter pTEFmut8, which has 70% of the native pTEF1 promoter activity. Adapted for the MoClo kit as type 2. Amplified from Addgene plasmid #124582 with oligos LT176.	Addgene plasmid #124582; http://n2t.net/addgene:124582;
RNA_2.6	Sequence in the GlyRNA aptamer biosensor responsible for sensing fructose-bisphosphate. Adapted for the MoClo kit as type 4a. Amplified from Addgene plasmid #162800 with oligos LT175.	Addgene plasmid #162800; http://n2t.net/addgene:162800;
3’ X2 homology Arm	100-bp oligo containing the sequence of the 3’ homology arm of the X2 site. Adapted for the MoClo kit as type 7. Amplified from template oligos LT171 with oligos LT177.	Oligos LT171
5’ X2 homology Arm	100-bp oligo containing the sequence of the 5’ homology arm of the X2 site. Adapted for the MoClo kit as type 8b. Amplified from template oligos LT172 with oligos LT178.	Oligos LT172
Dummy - type 6	Dummy sequence used to avoid integration of selection markers in the genome. Adapted for the MoClo kit as type 6. Amplified from oligos LT179.	Oligos LT179

Supplementary Table 3. Plasmids used in this study. List of plasmids developed in this study. The names in *italics* refer to plasmids used as donor DNA for yeast genome integration after linearisation.

Plasmid name	Description	REF	Addgene ID
YN2_1_Cas9_exp	Cas9 plasmid with scaffold sgRNA cassette and GFP dropout.	(Cámara et al., 2020)	--
YN2_1_LT58_X2site	Cas9 plasmid with sgRNA LT58 targeting the X2 site.	This Study	177705
YN2_1_LT84_RPL13A	Cas9 plasmid with sgRNA LT84 targeting the C-terminal region of RPL13A.	This Study	177712
LT1_33_pTEFmut8-mCherry	Normalisation construct (pTEFmut8-mCherry) with X2 homology arms.	This Study	177706
LT1_34_sfpHluorin	Intracellular pH biosensor (pTEF1-sfpHluorin) with X2 homology arms.	This Study	177707
LT1_36_QUEEN-2m	Intracellular ATP biosensor (pTEF1-QUEEN2m) with X2 homology arms.	This Study	177708
LT2_7_OxPro	Oxidative stress biosensor (pTRX2_5xUAS + ymYPET) + normalisation construct (LT1_33) with X2 homology arms.	This Study	177709
LT2_12_GlyRNA	Glycolytic flux biosensor (GlyRNA, pTEFmut8-mTurquoise2-RNA_2.6) + normalisation construct (LT1_33) with X2 homology arms.	This Study	177710
LT2_14_GlyOx	Glycolytic flux biosensor (GlyRNA, pTEFmut8-mTurquoise2-RNA2.6) + oxidative stress biosensor (pTRX2_5xUAS + ymYPET) + normalisation construct (LT1_33) with X2 homology arms.	This Study	177711

Supplementary Table 4. Oligos used in this study. List of the main oligos used in this study.

Oligo code	Description	Sequence (5' to 3')
LT57_F	Forward oligo for amplifying pTRX2_5xUAS from Addgene plasmid #124708 (uppercase) with restriction sites for pYTK001 (lowercase).	gcatcgtctcatcggtctcaaacgAACGGAATGCGTGCGATC
LT57_R	Reverse oligo for amplifying pTRX2_5xUAS from Addgene plasmid #124708 (uppercase) with restriction sites for pYTK001 (lowercase).	atgccgtctcaggtctcacataTGTTTTATGACAGATTATTGATGTG
LT58_F	Forward sgRNA for the X2 site (uppercase) with sticky ends for YN2_1_Cas9_exp (lowercase).	gactTGCATAATCGGCCCTCACAG
LT58_R	Reverse sgRNA for the X2 site (uppercase) with sticky ends for YN2_1_Cas9_exp (lowercase).	aaacCTGTGAGGGCCGATTATGCA
LT84_F	Forward sgRNA for tagging RPL13A (uppercase) with stick ends for YN2_1_Cas9_exp (lowercase).	gactGAAGGAAAATACAAAAATTG
LT84_R	Reverse sgRNA for tagging RPL13A (uppercase) with sticky ends for YN2_1_Cas9_exp (lowercase).	aaacCAATTTTTGTATTTTCCTTC
LT87_F	Forward oligo for verifying correct integration in the RPL13A C-terminal region.	AGGCCCCAGAAGCTGAACAAG
LT88_R	Reverse oligo for verifying correct integration in the RPL13A C-terminal region.	CCATCTTTCGCATCTCTTCTATGC
LT171_F	Forward 100-bp oligo containing the sequence of the 3' homology arm of the X2 site in *S. cerevisiae* CEN.PK113-7D.	GACGAAGGCTAAGGTCACTTCTTCGTTTCCTTTATTGGGGTTTCCGTGTAGCCTTCCCCTGAATAGTGTGGGACGTTTTATGAGAAGCCGTAAGAAATAG
LT171_R	Reverse 100-bp oligo containing the sequence of the 3' homology arm of the X2 site in *S. cerevisiae* CEN.PK113-7D.	CTATTTCTTACGGCTTCTCATAAAACGTCCCACACTATTCAGGGGAAGGCTACACGGAAACCCCAATAAAGGAAACGAAGAAGTGACCTTAGCCTTCGTC
LT172_F	Forward 100-bp oligo containing the sequence of the 5' homology arm of the X2 site in *S. cerevisiae* CEN.PK113-7D.	GTCAAAAGATCCTCTCATACCATATTAAGTAAATTGCCTCCATTTCTTTTTCCTCGGGCAGAGAAACTCGCAGGCAACTTGCTCTCGAAGTGGTCACGTG
LT172_R	Reverse 100-bp oligo containing the sequence of the 5' homology arm of the X2 site in *S. cerevisiae* CEN.PK113-7D.	CACGTGACCACTTCGAGAGCAAGTTGCCTGCGAGTTTCTCTGCCCGAGGAAAAAGAAATGGAGGCAATTTACTTAATATGGTATGAGAGGATCTTTTGAC
LT174_F	Forward oligo for amplifying mTurquoise2 and tagging the C-terminal region of RPL13A using pYTK032 (MoClo kit) (lowercase) with homology arms for RPL13A (uppercase).	AAGAGAGCTAGAGAAAAGGCTGAAGCTGAAGCTGAAAAGAAGAAAGGATCCgtttctaaaggtgaagaatta
LT174_R	Reverse oligo for amplifying mTurquoise2 and tagging the C-terminal region of RPL13A using pYTK032 (MoClo kit) (lowercase) with homology arms for RPL13A (uppercase).	CATATATATTATTTATGAAATTGAACAGATAAAAAAGAAGGAAAACTAtttgtacaattcatccatacc
LT175_F	Forward sequence sensing fructose-bisphosphate in GlyRNA (lowercase) with restriction sites for pYTK001 (uppercase).	GCATCGTCTCATCGGTCTCAATCCtaaagcctaggaaacaaacaaagctgtcaccggaataggagtccggtctgatgagtccacttgctgaggatccgacggttcc

LT175_R	Reverse sequence sensing fructose-bisphosphate in GlyRNA (lowercase) with restriction sites for pYTK001 (uppercase).	ATGCCGTCTCAGGTCTCAGCCActcgaggctcgagtttttatttttctttttgctgtttcgtccttcgtcaacacatgaggaaccgtcggatcctcagcaagtggactc
LT176_F	Forward oligo for amplifying pTEFmut8 from Addgene plasmid #124582 (lowercase) with restriction sites for pYTK001 (uppercase).	GCATCGTCTCATCGGTCTCAAACGatagcttcgaaacgtttctactc
LT176_R	Reverse oligo for amplifying pTEFmut8 from Addgene plasmid #124582 (lowercase) with restriction sites for pYTK001 (uppercase).	ATGCCGTCTCAGGTCTCACATAttttctagaaaacttagattagattgct
LT177_F	Forward oligo for amplifying the 3' homology region of the X2 site (uppercase) with restriction sites for pYTK001 (lowercase).	gcatcgtctcatcggtctcagagtGACGAAGGCTAAGGTCACT
LT177_R	Reverse oligo for amplifying the 3' homology region of the X2 site (uppercase) with restriction sites for pYTK001 (lowercase).	atgccgtctcaggtctcatcggCTATTTCTTACGGCTTCTCAT
LT178_F	Forward oligo for amplifying the 5' homology region of the X2 site (uppercase) with restriction sites for pYTK001 (lowercase).	gcatcgtctcatcggtctcacaatGTCAAAAGATCCTCTCATACCA
LT178_R	Reverse oligo for amplifying the 5' homology region of the X2 site (uppercase) with restriction sites for pYTK001 (lowercase).	atgccgtctcaggtctcaagggCACGTGACCACTTCGAGAG
LT179_F	Forward oligo for amplifying the dummy type 6 (uppercase) with restriction sites for pYTK001 (lowercase).	gcatcgtctcatcggtctcatacaTAACCCCTAGGTCTTCCTGA
LT179_R	Reverse oligo for amplifying the dummy type 6 (uppercase) with restriction sites for pYTK001 (lowercase).	atgccgtctcaggtctcaactcTCAGGAAGACCTAGGGGTTA
LT180_F	Forward sequence insensitive to fructose-bisphosphate in mutGlyRNA (uppercase) with restriction sites for pYTK001 (lowercase).	gcatcgtctcatcggtctcaatcctaaAGCCTAGGAAACAAACAAAGCTGTCACCGGAATAGGAGTCCGGTCTGATGAGTCCTGTTGCTGAGGATCCGACGGTTCC
LT180_R	Reverse sequence insensitive to fructose-bisphosphate in mutGlyRNA (uppercase) with restriction sites for pYTK001 (lowercase).	atgccgtctcaggtctcagccactcgaGGCTCGAGTTTTTATTTTTCTTTTTGCTGTTTCGTCCAAGCAGTTCACATGAGGAACCGTCGGATCCTCAGCAACAGGACTC
LT181_F	Forward oligo for verifying correct integration in the HO site.	ATTCATTCACATCATTTTCGTGGATCC
LT181_R	Reverse oligo for verifying correct integration in the HO site.	GTGCCTTTGGACTTAAAATGGCG
LT183_F	Forward oligo for verifying the presence and correct integration in the X2 site.	TGCTCGATCTTCTATCCTCTTTAGG
LT183_R	Reverse oligo for verifying the presence and correct integration in the X2 site.	AAGTTCTTTGTAGAACAGCTCTCTC
LT185_F	Forward oligo for sequencing the X2 site.	GATATCAACTACGAGAGCGATCG
LT185_R	Reverse oligo for sequencing the X2 site.	GGAACAGATTTACGCGTTTAGGATG

Supplementary Table 5. Fluorescence filters used in this study. List of filters and gains used in this study for fluorescence detection in the BioLector I. All filters are 10-nm bandpass for both excitation (ex) and emission (em) wavelengths.

Fluorescent protein	Filter code	Filter details
mTurquoise2	E-OP-309	ex436/em488 nm, gain = 50
ymYPET	E-OP-315	ex508/em532 nm, gain = 55
mCherry	E-OP-319	ex580/em610 nm, gain = 60
sfpHluorin / QUEEN-2m	E-OP-341	ex400/em510 nm, gain = 20
	E-OP-304	ex488/em520 nm, gain = 40

2.2. Supplementary Figures

Supplementary Figure 1. Sequence comparison of the X2 site (next page). Sequencing alignment of the X2 site across different *Saccharomyces cerevisiae* strains and *Saccharomyces boulardii* CNCM I-745 (“PRECOSA”). Laboratory strains are in bold, industrial strains are in bold and italics, and wild-type strains are in italics. Nucleotides in green refer to single nucleotide polymorphisms, nucleotides in red represent deletions, and nucleotides in yellow represent insertions. The sequence targeted by the sgRNA is enclosed by a blue square. Homology arms used for genome integration are enclosed by black squares. Oligos LT183-F and LT193_R used for colony PCR to verify correct integration are enclosed by purple squares.

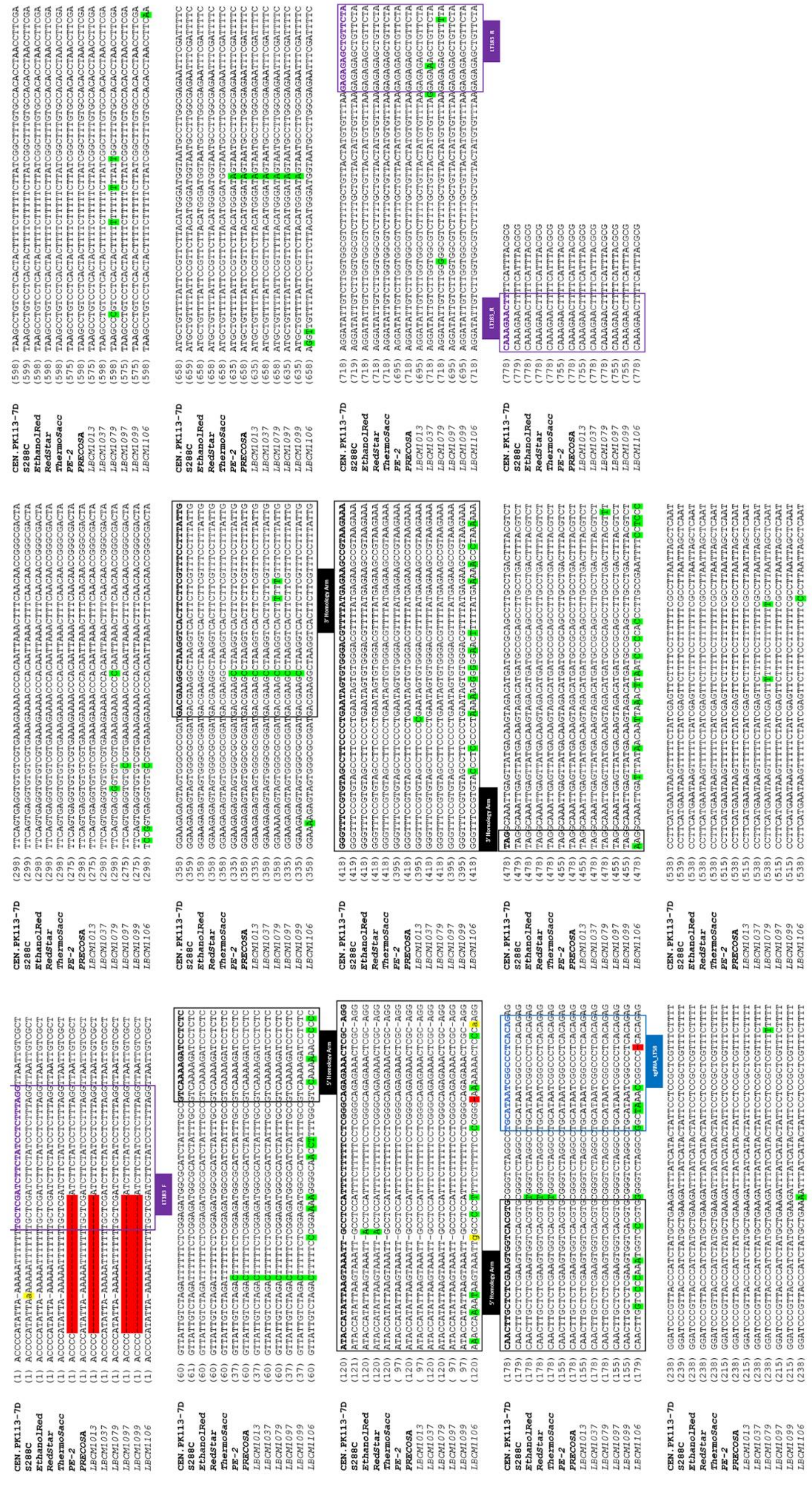

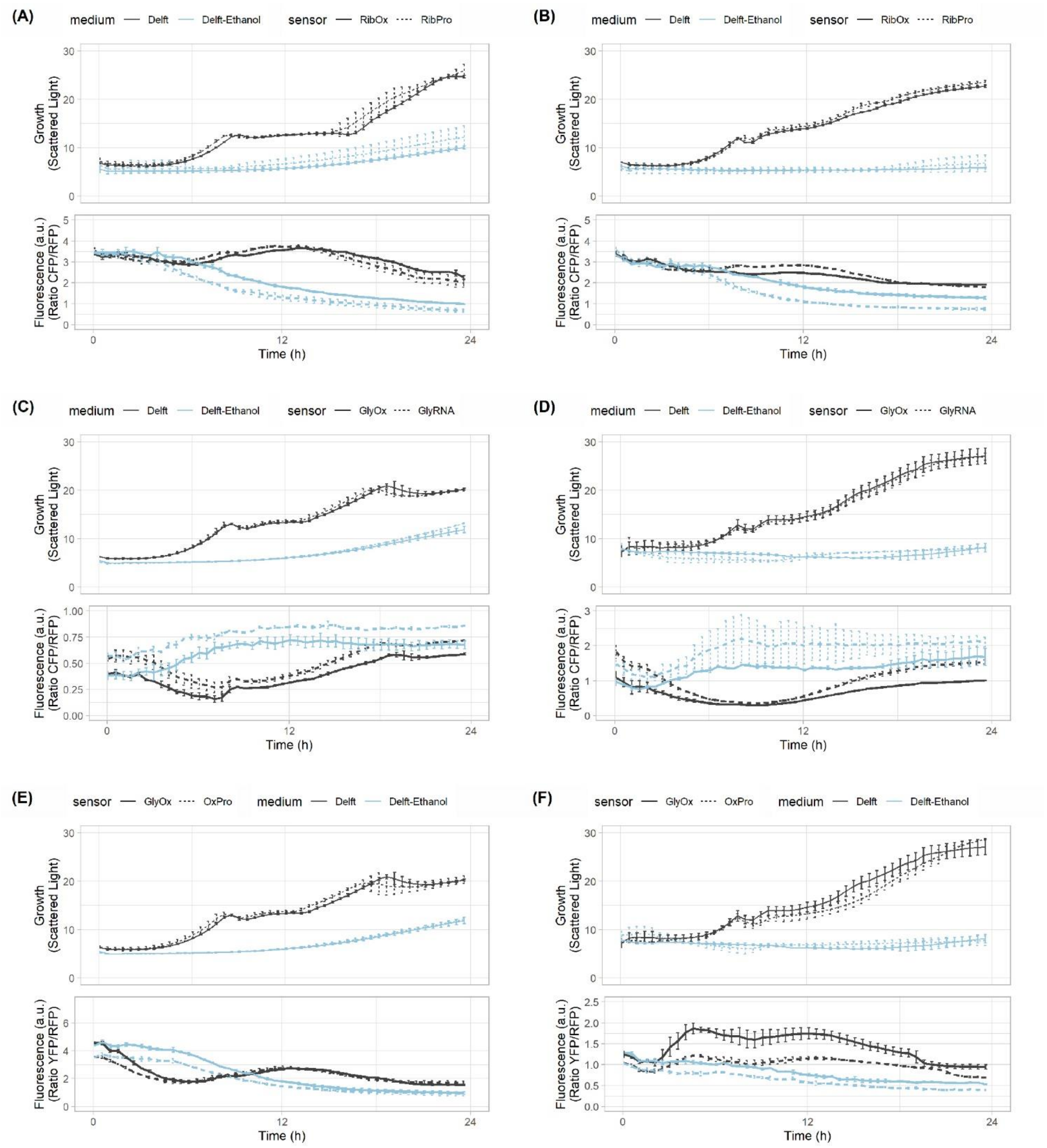

Supplementary Figure 2. Growth and fluorescence curves comparing strains harbouring single or multiple biosensors grown on different carbon sources. (A, C, E) CEN.PK113-7D and (B, D, F) Ethanol Red yeast strains were cultured in Delft medium supplemented with 20 g/L glucose (black lines) or 20 g/L ethanol (blue lines). Strains containing single (continuous lines), or multiple (dashed lines) biosensors were used to detect (A, B) ribosome production, (C, D) glycolytic flux, and (E, F) oxidative stress.

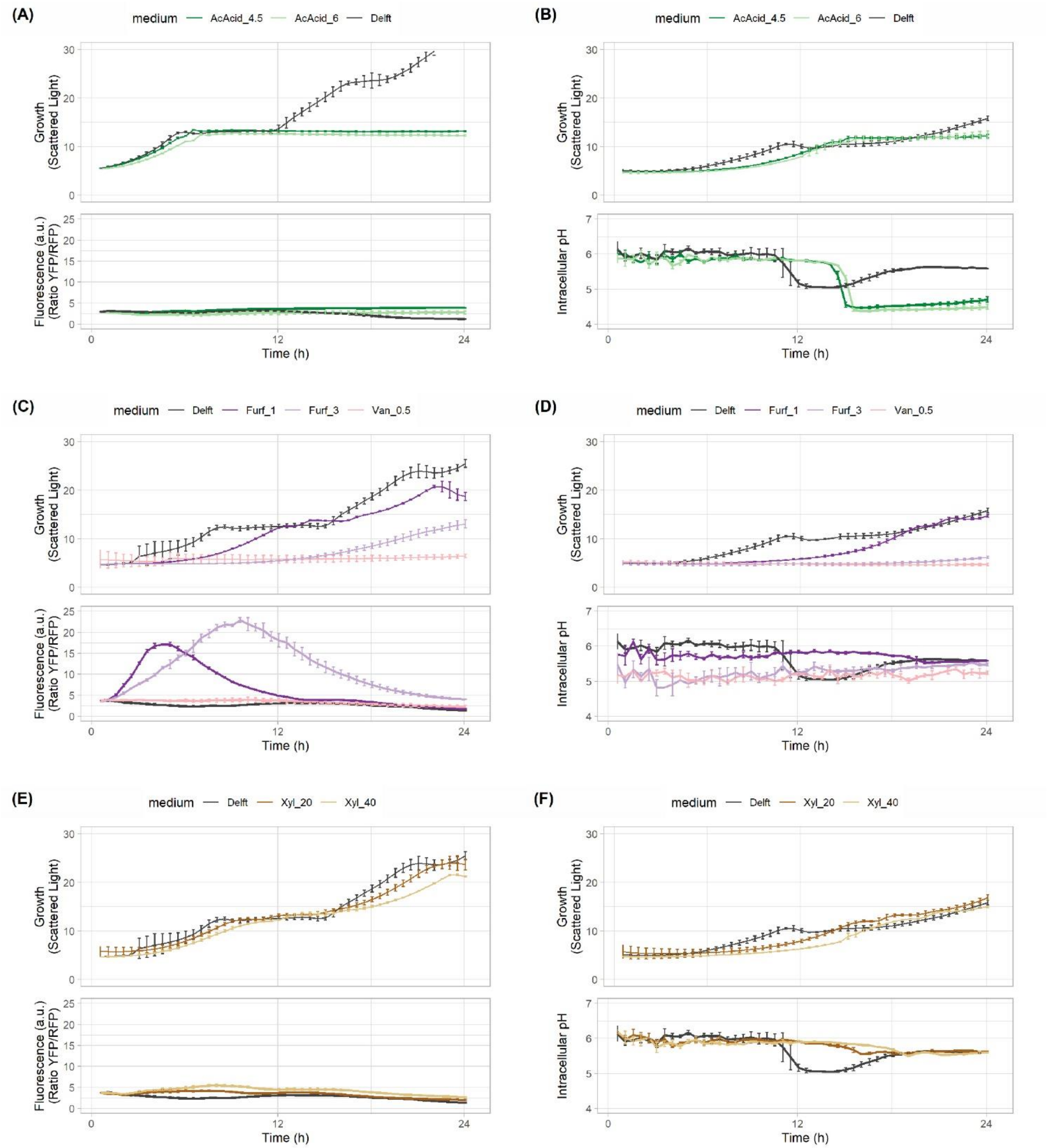

Supplementary Figure 3. Growth and fluorescence curves comparing oxidative stress and intracellular pH in CEN.PK113-7D yeast strains challenged with different lignocellulosic inhibitors. CEN.PK113-7D yeast strains were grown in the presence of (A, B) acetic acid (AcAcid) at 4.5 or 6 g/L, (C, D) furfural (Furf) at 1 or 3 g/L or vanillin (Van) at 0.5 g/L, and (E, F) xylose (Xyl) at 20 or 40 g/L. (A, C, E) Oxidative stress response activation and (B, D, F) intracellular pH were assessed using the biosensors OxPro and sfpHluorin, respectively.

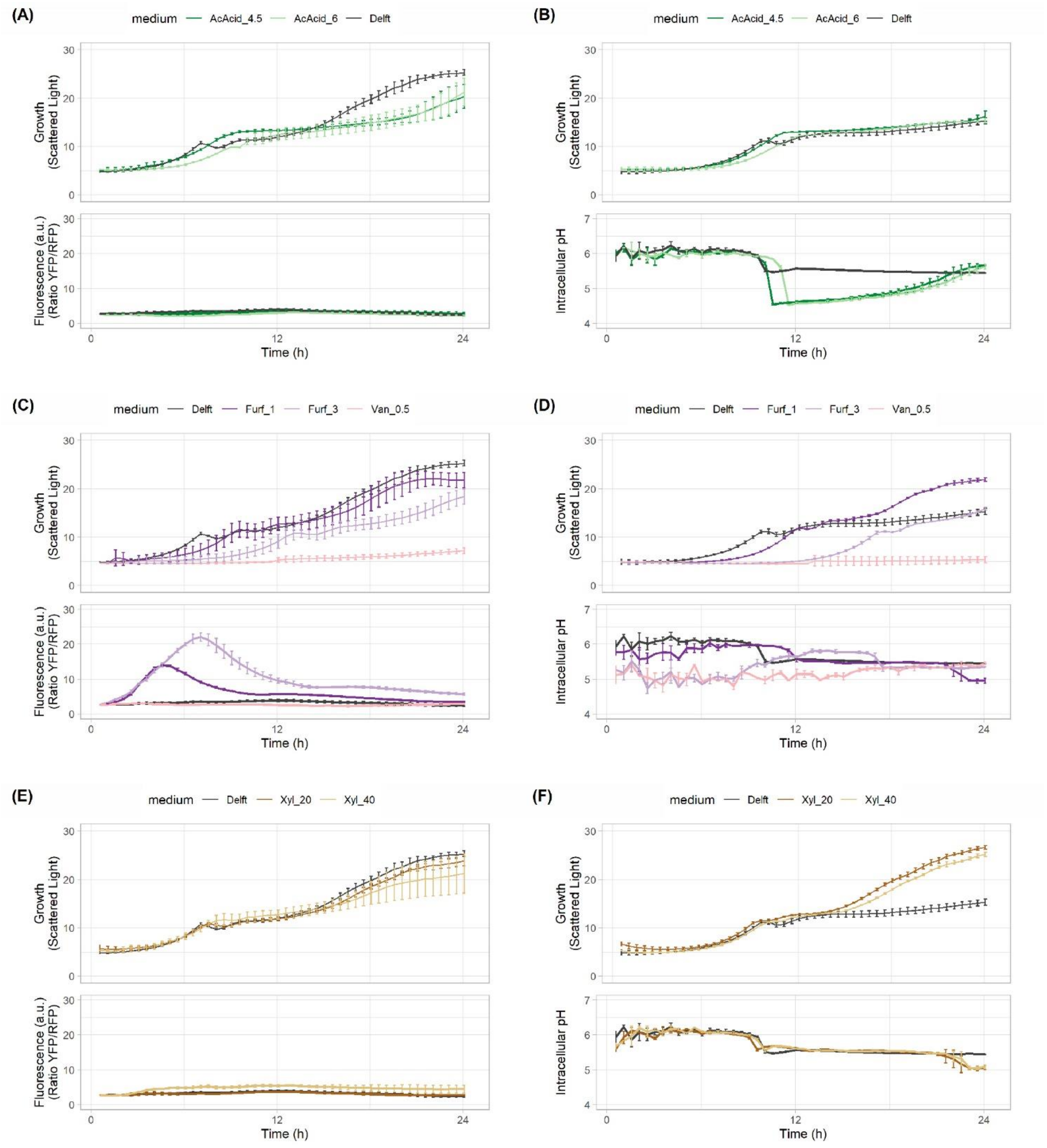

Supplementary Figure 4. Growth and fluorescence curves comparing oxidative stress and intracellular pH in Ethanol Red yeast strains challenged with different lignocellulosic inhibitors. Ethanol Red yeast strains were grown in the presence of (A, B) acetic acid (AcAcid) at 4.5 or 6 g/L, (C, D) furfural (Furf) at 1 or 3 g/L or vanillin (Van) at 0.5 g/L, and (E, F) xylose (Xyl) at 20 or 40 g/L. (A, C, E) Oxidative stress response activation and (B, D, F) intracellular pH were assessed using the biosensors OxPro and sfpHluorin, respectively.

3. Supplementary References

Botman, D., de Groot, D.H., Schmidt, P., Goedhart, J., Teusink, B., 2019. In vivo characterisation of fluorescent proteins in budding yeast. Sci. Rep. 9.

Cámara, E., Lenitz, I., Nygård, Y., 2020. A CRISPR activation and interference toolkit for industrial Saccharomyces cerevisiae strain KE6-12. Sci. Rep. 10.

Elsutohy, M.M., Chauhan, V.M., Markus, R., Kyyaly, M.A., Tendler, S.J.B., Aylott, J.W., 2017. Real-time measurement of the intracellular pH of yeast cells during glucose metabolism using ratiometric fluorescent nanosensors. Nanoscale 9, 5904–5911.

Guldfeldt, L.U., Arneborg, N., 1998. Measurement of the Effects of Acetic Acid and Extracellular pH on Intracellular pH of Nonfermenting, Individual Saccharomyces cerevisiae Cells by Fluorescence Microscopy. Appl. Environ. Microbiol. 64, 530.

Lambert, T.J., 2019. FPbase: a community-editable fluorescent protein database. Nat. Methods 2019 164 16, 277–278.

Nevoigt, E., Kohnke, J., Fischer, C.R., Alper, H., Stahl, U., Stephanopoulos, G., 2006. Engineering of Promoter Replacement Cassettes for Fine-Tuning of Gene Expression in Saccharomyces cerevisiae. Appl. Environ. Microbiol. 72, 5266.

Rowe, S.M., Simpson, W.J., Hammond, J.R.M., 1994. Intracellular pH of yeast during brewery fermentation. Lett. Appl. Microbiol. 18, 135–137.
